# Antimicrobial and anti-cancer potential of turmeric synthesized AuNPs and Chitosan-AuNP nanocomposites against MDR pathogens and breast/colorectal carcinoma cells

**DOI:** 10.1186/s13568-025-01953-y

**Published:** 2025-11-19

**Authors:** Mokhtar Saeed Rejili, Faouzi Haouala, Ahmed M. Abdulfattah, Ahmad F. Alhomodi, Majid Al-Zahrani, Sawsan Abd Ellatif, Elsayed E. Hafez, Elsayed S. Abdelrazik

**Affiliations:** 1 Department of Life Sciences, College of Sciences, Al Imam Mohammad Ibn Saud Islamic University (IMSIU), Riyadh, 11623 Saudi Arabia; 2 Department of Medical Laboratory Sciences, Faculty of Applied Medical Sciences, King Abdulaziz University, Jeddah, 21589 Saudi Arabia; 3 Department of Biology, College of Science and Arts, Kingdom of Saudi Arabia, Najran University, Najran, Saudi Arabia; 4 Department of Biological Sciences, Faculty of Sciences and Arts, King Abdulaziz University, Rabigh, Saudi Arabia; 5 Bioprocess Development Department, Genetic Engineering and Biotechnology Research Institute (GEBRI), City of Scientific Research and Technological Applications (SRTA-City), New Borg El-Arab City, P.O. 21934, Alexandria, Egypt; 6 Plant Protection and Bimolecular Diagnosis Department, Arid Lands Cultivation Research Institute (ALCRI), City of Scientific Research and Technological Applications (SRTA-City), New Borg El-Arab City, P.O. 21934, Alexandria, Egypt; 7 Livestock Research Department, Arid Lands Cultivation Research Institute (ALCRI), City of Scientific Research and Technological Applications (SRTA-City), New Borg El-Arab City, P.O. 21934, Alexandria, Egypt

**Keywords:** Virulent gene, Chitosan-turmeric gold nanocomposite, Anti-biofilm, Antioxidants, Anticancer, Characterization

## Abstract

The rise in antimicrobial resistance to current antibiotics has driven the search for novel natural compounds with antimicrobial properties. This study aimed at evaluate the Antimicrobial, Anti-Biofilm, and Cytotoxic Potential of Turmeric synthesized AuNPs and Chitosan-AuNP Nanocomposites against MDR urinary tract infections (UTIs) Pathogens (*K. pneumoniae* and *E. coli*) and MCF-7 & HCT-116 Cancer Cell Lines. Biosynthesize- characterization of turmeric-gold nanoparticles (TAuNPs) using methanolic extract of *Curcuma longa* rhizome by Transmission Electron Microscopy (TEM), X-ray spectroscopy, Fourier Transform Infrared (FT-IR) spectroscopy, and Zeta potential (Z-potential) analysis. Coated biosynthesized TAuNPs with biodegradable chitosan to form a chitosan-turmeric gold nanocomposite (NCS-TAuNPs). Phytochemical and gas chromatography-mass spectrometry (GC–MS) analysis were used to investigate the total phenolic and flavonoids contents, and chemical properties of the *C. longa* extract, assessment the antimicrobial and anti-biofilm efficiency of TAuNPs and NCS-TAuNPs conjugate against *K. pneumoniae* ESA254 and *E. coli* ESA253 isolates, evaluation in vitro, the cytotoxicity effects of TAuNPs and NCS-TAuNPs conjugate against MCF-7 cells and HCT-116 cancer cell lines copared with Fluorouracil (5FU) chemical anticancer drug. The results indicated that, presence of high levels (3452 mg QE/100 g DW and 2624 mg GAE/100 g DW) of flavonoids and phenolic contents in *C. longa* extract, the antioxidant scavenging abilities of 150 μg/mL of TAuNPs and NCS-TAuNPs conjugate against ABTS free radicals were reported 63.72 ± 0.09% and 52.55 ± 0.07% respectively, and 50.29 ± 0.038% and against DPPH free radicals were 68.43 ± 0.095% respectively. The maximum antibacterial activity by inhibiting *K. pneumoniae* ESA254 and *E. coli* ESA253 were recorded 15.6 ± 0.2 mm and 13.3 ± 0.20 mm at exposed to 150 µg/mL of NCS –TAuNPs, 14.3 ± 0.3 mm and 10.8 ± 0.23 mm by exposed to 150 µg/mL TAuNPs respectively. The maximum anti-biofilm activity (biofilm reduction %) of *K. pneumoniae* ESA254 and *E. coli* ESA253 were recorded 85.67 ± 2.46% and 77.50 ± 2.8% at exposed to 150 µg/mL of NCS –TAuNPs, 63.82 ± 1.89% and 58.07 ± 2.13% by exposed to 150 µg/mL TAuNPs respectively. The anticancer activity of TAuNPs and NCS–TAuNPs conjugate against MCF-7 and HCT-116 cell lines as cell viability %, showed potential cytotoxic effect in MCF-7 cells with IC_50_ values 38.77 ± 3.1 μg/mL and 36.31 ± 2.9 μg/mL exposed to 150 μg/mL of NCS–TAuNPs and TAuNPs respectively, and 41.26 ± 1.9 μg/mL and 39.85 ± 2.7 μg/mL in HCT-116 cell exposed to 150 μg/mL of NCS–TAuNPs and TAuNPs respectively. The study concluded that, the resulting biosynthesized TAuNPs and NCS–TAuNPs nanocomposite exhibited novel potential dual- anaotherapeutic efficiency combating MDR UTI pathogens and cancer treatment, the safe and biocompatible chitosan nanoconjugate is drug carriers with intelligent therapeutic platform, offering synergistic efficacy to AuNPs as anticancer and antibacterial agents against MCF-7 and HCT-116 cancer cell lines, against infections caused by *K. pneumoniae* and *E. coli* pathogens.

**Graphical abstract:**

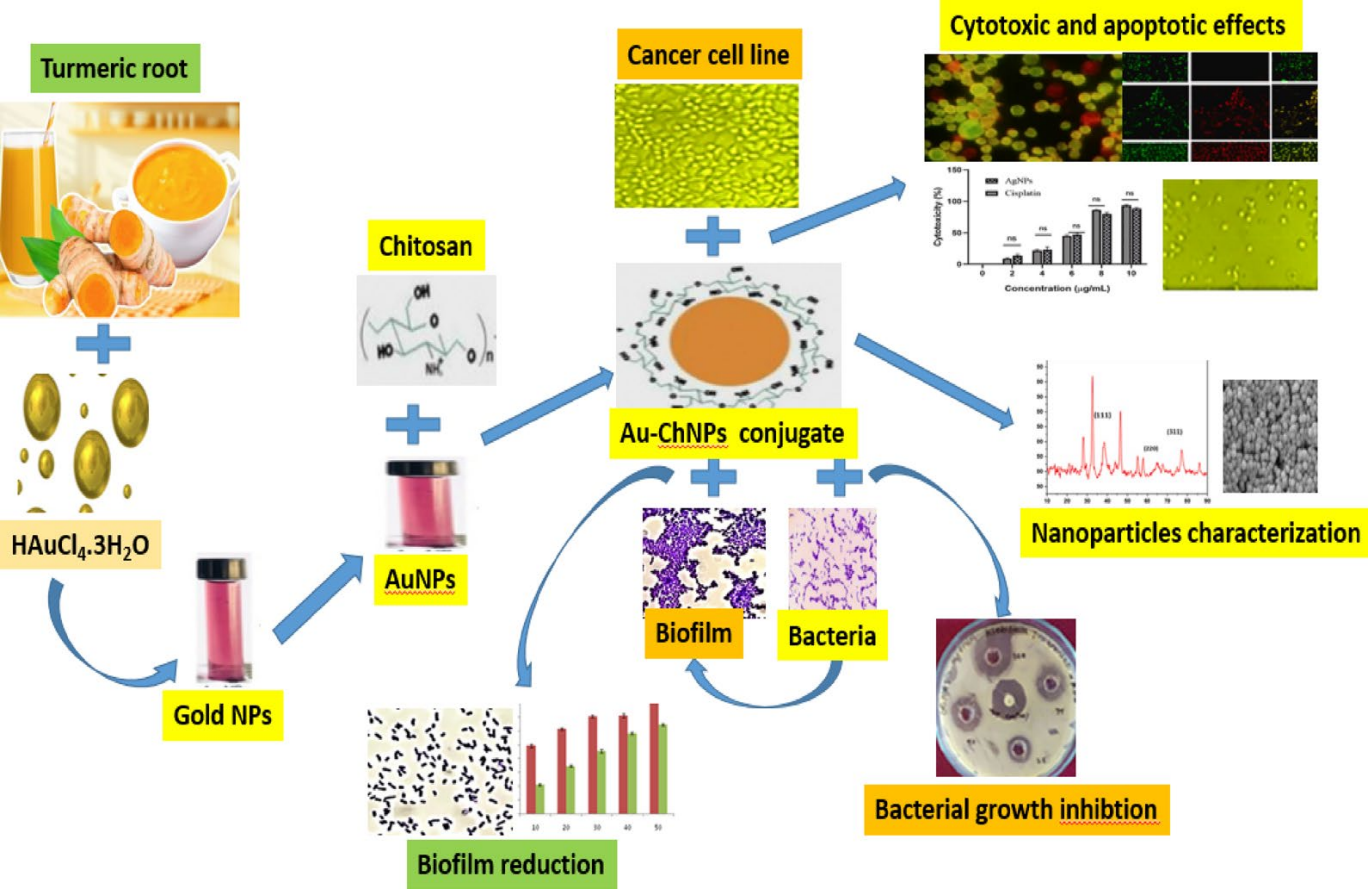

**Supplementary Information:**

The online version contains supplementary material available at 10.1186/s13568-025-01953-y.

## Introduction

The practice of using medicinal plants for healing dates back to ancient times and remains a cornerstone of traditional healthcare systems worldwide for various reasons (Abdallah et al. 2023). The development of effective medicinal products can be further advanced by modifying the chemical composition, biological functions, and medicinal properties of herbs (Wang et al. 2023).

In recent years, a lot of interest has been paid to metallic nanoparticles because of their special physico-chemical characteristics, and broad range of applications. Among these, chitosan-coated gold nanoparticles have emerged as promising bioactive hybrid materials due to their biodegradability, biocompatibility, high activity, stability, and low toxicity (Kumar-Krishnan et al. 2015; Fuster et al. 2020; Mais et al. 2025). Chitosan, a biopolymer known for its non-toxic nature, exhibits unique characteristics such as small size, surface effects, interface properties, and quantum size effects. Additionally, it possesses exceptional antibacterial and antifungal properties against a wide range of pathogenic microorganisms (Fuster et al. 2020**;** Mais et al. 2025a). As a natural polysaccharide, Chitosan receives extensive application in the food and medicinal sectors due to its high biocompatibility, biodegradability, and low toxicity (Abdelhamid et al. 2019; Panda et al. 2022). Furthermore, chitosan serves as an effective stabilizing agent in the synthesis of various metallic nanoparticles (Corbierr et al. 2004; Chompooso et al. 2008; DeLong et al. 2010; Hashem et al. 2022). Chitosan-guaranteed nanoparticles have attracted significant attention due to antifungal, antibacterial, antiprotozoal, anticancer, antiplaque therapeutic efficiency (Sharifiaghdam et al. 2021; Mais et al. 2025a). The CS/Fe3O4 NPs were found to be less cytotoxic yet exhibit great antibacterial efficiency against both planktonic bacteria and their biofilms (Mais et al. 2025b). Furthermore, *Artemisia pallens* synthesized MnNPs exhibited antibacterial and anti-biofilm efficacy against *S. aureus*, *S. mutans* and *P. aeruginosa* (El-Tayeb et al. 2025). Pathogenic microbes often form biofilms composed of exopolysaccharides, which act as protective barriers, shielding them from harsh conditions such as disinfectants and antibiotics (Abdallah et al. 2014). Antimicrobial resistance (AMR) poses a significant and growing threat to global health, happened when microorganisms categorized as multidrug-resistant (MDR) shift to resistant against current antibiotics and chemotherapeutic medications (Mostafa et al. 2022). To date, specific resistance mechanisms have emerged against the majority of antibiotics, highlighting the urgent need for innovative and effective antibacterial strategies to combat this escalating challenge (Hassan and Hamed 2022).

The current study aimed to synthesize green turmeric gold nanoparticles (TAuNPs) using methanolic extract of *Curcuma longa* and to develop biodegradable chitosan-coated gold nanoparticle conjugates (NCS-TAuNPs) with enhanced bioactivity. The novelty of this work lies in both the eco-friendly synthesis approach, combining the therapeutic properties of turmeric and chitosan to produce biocompatible nanocomposites. Also, innovative nanocomposite design production in the combination of turmeric-synthesized gold nanoparticles with chitosan creates a novel nanocomposite that leverages the biocompatibility of chitosan with the therapeutic properties of both gold nanoparticles and turmeric compounds. The TAuNPs nanoparticles were comprehensively characterized and evaluated the antioxidant, antibacterial, and anti-biofilm activities of TAuNPs and NCS-TAuNPs against multidrug-resistant *K. pneumoniae* and *E. coli*. Additionally, In vitro, cytotoxic effects of TAuNPs and NCS-TAuNPs nanocomposite were assessed against breast adenocarcinoma (MCF-7) and colorectal (HCT-116) cell lines, demonstrating their potential as natural alternatives to conventional antimicrobial and anticancer treatments.

## Materials and methods

The amplified PCR product of 16S gene from bacterial isolates were sent for sequencing using the specific 16S forward primer, Sequencing was accomplished using ABI PRISMTM 3100 DNA sequencer (Applied Biosystems) and Big Dye terminator sequencing kit (Version 3.1, Applied Biosystems, USA), The obtained sequences were first edited and subjected to BLAST search to assign putative identity with similar sequences using NCBI database http://blast.ncbi.nlm.nih.gov/Blast.cgi. Sequences were then aligned with other similar sequences already present in GenBank and downloaded from GenBank using ClustalX (Thompson et al. 2018) and Molecular Evolutionary Genetics Analysis (MEGA) software ver. 11.0 (Tamura et al. 2011), nucleotide sequence of the pathogenic bacteria were identified as *K. pneumoniae* strain ESA254 and *E. coli* strain ESA253, and have been submitted to GenBank for accession number PV017749 and PV017748 respectively.

### Biochemical characterization of isolated bacteria

Standard techniques for biochemical assays were followed in the biochemical profiles of the UTIs bacterial isolates; *K. pneumonia* ESA254 and *E. coli* ESA253, and ATCC reference bacterial strains; *K. pneumoniae* ATCC13883 and *E. coli* ATCC 25922 strains. (Catalase, oxidase, methyl red, citrate, urease, indole production and Voges-Proskauer tests).

### Disk diffusion susceptibility assay

The bacteria's antibiotic susceptibility testing was conducted on UTIs bacterial isolates; *K. pneumonia* ESA254 and *E. coli* ESA253 isolates, and ATCC reference bacterial strains; *K. pneumonia* ATCC13883 and *E. coli* ATCC 25922 strains using the disc diffusion method of the Kirby-Bauer antibiotic assay. To put it briefly, isolates of *K. pneumoniae* and *E. coli* were streaked onto LB agar plates and cultured for 24 h at 37 ˚C. Utilizing a UV–Vis spectrophotometer (Genway, Japan) at 625 nm, the optical density (OD) of the bacterial suspensions was measured and adjusted to 1.5 × 10^8^ colony forming units/mL by 0.9% (w/v) saline solution according to ( European Committee on Antimicrobial Susceptibility Testing 2022**,** Mais et al.2025c). Bacterial suspension (100 µL) was streaked on LB agar plate, add fixed concentration of different antibiotics on filter paper discs that placed on the surface of the agar media and incubated at 37 ˚C for 24 h in triplicate manner, plates were After incubation, the diameter of the zone of inhibition around each antibiotic disc was measured to the closest mm and compared to Clinical and Laboratory Standards Institute (CLSI 2022) standard.

### Plant material and extraction

Fresh rhizomes of turmeric (*Curcuma longa*) were obtained from a local market (El-Hawag Maady Company, Alexandria, Egypt). A sample of turmeric rhizome was thoroughly ground with an electrical grinding machine, then left to dry in the shade. In a soxhlet extraction, 20 g of powdered material was added to 100 mL of hydro-alcoholic (50:50, V/V) water and methanol, the mixture was refluxed for around 12 h at 50 ˚C. The extracts were frozen at -80 ˚C after the extraction period, filtered twice with Whatman no. 1 filter paper, and then lyophilized. The percentage of extraction yield was determined using the following formula (Elizondo-Luevano et al. 2020**)**:

$$ \% {\text{ Yield }} = \frac{{{\text{Final weight}} }}{{\text{Initial weight}}} \times { 1}00 $$

### Qualitative phytochemicals

The hydro-alcoholic extracts of turmeric rhizome was dissolved in 1.0% dimethylsulfoxide (DMSO) and then screened for the presence of various secondary metabolites and bioactive components, such as tannins, steroids, saponins, phenols, flavonoids, terpenoids, and glycosides, according to previously described methods (Blažeković, et al. 2010; Punasiya, et al. 2020; Iqbal, et al. 2015**).**

### Total phenolic content (TPC)

Total polyphenols in methanolic turmeric root extract were determined using the Folin-Ciocalteu reagent procedure described by Jain, et al. (2015). 0.3 mL of algal extract was blended with 75 µL of Folin-Ciocalteu and 5 mL of distilled water. The solution was incubated for 30 min at 25 °C and then 0.5 mL of 7.5% Na_2_CO_3_ and additional distilled water were added. Samples were incubated in a dark place at room temperature for 90 min. Gallic acid standard solutions of concentrations 25, 50, 75, 100, 125, and 150 µg/mL were prepared. The sample absorbance was recorded against a blank at 760 nm by using UV–Vis Spectrophotometer (Jenway, Japan). Total polyphenols content was estimated from the standard curve and expressed in the form as mg GAE/G. DW of extract.

### Total flavonoid content (TFC)

Total flavonoid content (TFC) of turmeric root extract was determined by Chang’s method (Chang, et al. 2002**).** To 200 μL of extract, 1.0 mL of methanol, 0.5 mL of distilled water, 50 μL of 10% aluminum chloride, and 50 μL of 1 M potassium acetate were added in a test tube. The reaction mix was kept in dark for 30 min. The absorbance reading of the reaction mix was recorded at 415 nm on UV–Vis spectrophotometer (Jenway, Japan). The total flavonoid content was determined from a standard curve and expressed in milligrams of quercetin equivalent per gram of extract (mg QE/g DW).

### Gas chromatography–mass spectrometry (GC–MS)

The chemical constituents of the methanolic extract of turmeric was examined using gas chromatography–mass spectrometry (GC–MS SCION 456). The following operating conditions were used: column SOLGEL-WAX, 60 m × 0.25 mm i. d., thickness of the film: 0.25 μm, carrier gas: nitrogen regulated to the current of 1 mL/min, the temperature of injection to the detector FID: 220 °C, respectively, 250 °C. Constituents of essential oil were determined by the time of their release (in minutes), and the obtained values were compared to the literature and the results were compared by using MSWS 8 software, Automated Mass Spectral Deconvolution and Identification System (AMDIS) for GC–MS and NIST library.

### Gold nanoparticle biosynthesis applying extract of Curcuma longa

The green synthesis of turmeric-gold nanoparticles (TAuNPs) were synthesized according to Yadi et al. (2022) protocol. Briefly, 10 ml of 1 mM aqueous gold solution was prepared from of gold (III) chloride trihydrate (HAuCl_4_.3H_2_O) using de-ionized water and brought to a boil. Then, add equal volume of the turmeric extract to the gold solution and incubated in a stirring water bath at 600 rpm and 40 °C for 30 min. Finally, the dark red solution was developed indicating the formation of biogenic turmeric-gold nanoparticles. The suspension was filtered and left to dry at 50 °C for 24 h.

### Characterization of turmeric-gold nanoparticles

#### UV-vis absorption spectrophotometer

UV–Vis Absorption Spectrophotometer samples containing the created TAuNPs were subjected to measurements via UV–Vis absorption spectrophotometry with a 1 cm path length quartz cuvette using a UV–Vis spectrophotometer (Genway, Japan).

#### TEM and energy-dispersive x-ray spectroscopy

Transmission electron microscope equipment was used to analyze the surface morphology, size, shape, and distribution of the biosynthesized TAuNPs, which were collected on carbon-coated copper grids, using a JEM-2100F transmission electron microscope (JEOL, Tokyo, Japan) at a voltage of 200 kV (Sharma 2005). To analyze the elemental composition of the biosynthesized TAuNPs composite, a liquid nitrogen-cooled lithium-doped silicon EDX detector was used to detect energy-dispersive X-ray spectra. EDX analysis was performed using a Bruker system to identify the elemental composition and purity of the AuNPs (Goldstein et al. 2017).

#### Fourier-transform infrared spectroscopy (FTIR)

The functional groups and chemical structure of the phyto-compounds linked to the biosynthesized TAuNPs were investigated using a Bruker FTIR Tensor 27 spectrometer, located in Yokohama, Japan. The analysis was done within the range of 4000 to 400 cm^−1^, using a resolution of 4 cm^−1^ to reveal the bio-functional groups on the TAuNPs' surface (Colthup, **2012**).

#### Particle size analysis

Analysis of TAuNPs powder was carried out using a Zeta potential analyzer: the Malvern Zetasizer Nano ZS system from England. The equipment determines particle size distribution at 630 nm using a helium–neon laser. Freshly prepared gold solution was used. Synthesis of chitosan coated turmeric-gold Nanoparticles bio-conjugate.

### Synthesis of chitosan coated turmeric-gold Nanoparticles bio-conjugate

The synthesis of nano-chitosan coated turmeric-gold Nanoparticles (NCS-TAuNPs) conjugate according to Shao et al. (2022); Al-Sarraj et al. (2023) protocols with some modifications. Briefly, a Chitosan solution (0.2% w/v) was prepared by dissolving chitosan (commercial grade, Showa, Japan) in 1% w/v acetic acid solution (Sigma Aldrich, USA), then ultrasonically dispersed and dropped into acetic acid solution to final concentration 2.0% (w/v) under vigorous stirring for 1 h at 30 °C. Biogenic TAuNPs were added in a concentration of 10% (w/v), drop by drop per up to 20 mL chitosan solution and stirred continuously at 120 rpm for 8 h and 30 °C, dropwise 1 ml of 0.1% Tripolyphosphate (TPP) cross-linker for ionotropic gelation. Finally, the mixture’s color changed from slightly light yellow to dark wine-red indicating the synthesis Chitosan coated turmeric-gold nanoparticles conjugate with complete reduction and homogenization. The suspension that followed was filtered and dried in a vacuum after being washed three times with distilled water, poured into a sterile petri plate and incubated in a hot air oven at 50 °C for 24 h.

### Biological properties of TAuNPs and NCS –TauNPs conjugate

#### Antioxidant activity of TAuNPs and NCS –TAuNPs conjugate

The DPPH scavenging activities of various concentrations (50, 100, and 150 µg/ml) of TAuNPs and NCS –TAuNPs conjugate dissolved in 1.0% DMSO were assessed according to Chang et al. (2001) protocol, and the changes in the absorbance were measured at 517 nm via a UV‒Vis spectrophotometer (Jenway, Japan). The ABTS scavenging activities of the TAuNPs and NCS –TAuNPs conjugate dissolved in 1.0% DMSO were estimated at various concentrations (50, 100, and 150 µg/mL) according to (Casrto et al. 2021) protocol, and the DPPH and ABTS scavenging results are presented as IC_50_ values (µg/mL).

#### Antibacterial activity of TAuNPs and NCS –TAuNPs conjugate

Antibacterial activity of TAuNPs and NCS –TAuNPs conjugate against two urinary tract infection Gram-negative bacteria; *K. pneumoniae* ESA254 and *E. coli* ESA253 pathogens were assessed in vitro using different concentrations of TAuNPs and NCS –TAuNPs composites by agar diffusion methods according to US CLSI (Clinical and Laboratory Standards Institute, 2015) protocol, the TAuNPs and NCS-TAuNPs conjugate were dissolved in 0.5% dimethyl sulfoxide (DMSO, Sigma-Aldrich, USA) in 1 ml of DMSO to achieve the ideal quantities of nanoparticles (50, 100, and 150 µg/mL). The bacterial strains were cultured for two days at 37 °C on LB broth medium. After LB plates were prepared, 20µL of bacterial pathogens (about 10^5^ CFU/mL) were added. Sterile filter paper discs (5 mm in diameter) were subsequently immersed in varying quantities of nanoparticles (50, 100, and 150 µg/mL) as well as standard antibiotics (5 µg/mL) such as Augmentin and gentamicin once they had settled. For every treatment, the plates were tested in triplicate, incubated at 37 °C for 24 h. Following the incubation period, a ruler was used to measure the diameter of the clear zone surrounding the agar wells in millimeters (mm). Through a series of microdilutions, the minimal inhibitory concentration (MIC) was detected.

### Crystal violet assay for the determination of biofilm formation

#### In vitro biofilm formation

Briefly, Single-species biofilms were initiated by inoculating a 10^7^ CFU mL^−1^ bacterial suspension of *K. pneumoniae* ESA254 and *E. coli* ESA253 pathogens into 96-well tissue culture plates on 190-μL sterile LB broth media per well at 620 nm corresponds to an optical density (OD) of 0.13 (*K. pneumoniae*), and *E. coli* at 0.16 OD, with 3 replicate wells set up as a negative control with 200-μL sterile LB broth, then incubated at 37 °C for 24 h or 48 h under anaerobic conditions (Castro et al. 2021) and then washed 3 times with 200 μL of sterile distilled water, next, fixation with 200 μL of 100% (v/v) methanol for 20 min and gently decanted to remove free planktonic bacterial cells and the wells were washed three times with 200 μL of PBS buffer and air-dried for 60 min. These assays were repeated at least three times on separate days.

#### Crystal violet assay for quantification of biofilm formation

To quantify biomass ESA253 single species biofilms of *K. pneumoniae* ESA254 and *E. coli* ESA253 strains as per the crystal violet (CV) method of Peeters et al. (2008). In brief, after fixation step with methanol, biofilms were stained in 200 μL CV solution at 1% (v/v) (Sigma-Aldrich, Germany) for 20 min. All wells were washed 3 times, and dried at room temperature, washed with 200 μL of 33% (v/v) acetic for 10 min to completely dissolve the crystalline violet. For the estimation of total biofilm biomass, 3 measurements were taken at 620 nm of the solution produced by this using an enzyme marker, and the ODc value (mean OD of negative control + 3 × standard deviation) was taken as the cut-off value. Biofilm is said to be formed if the ODc value is higher than this. According to Devanga et al. (2020) the ability of strains to form biofilms could be classified into 4 grades: OD ≤ ODc as negative ( −), ODc < OD ≤ 2ODc as weak positive ( +), 2ODc < OD ≤ 4ODc as moderate positive (+ +), and OD > 4ODc as strong positive (+ + +).

### Sample collection and preparation

Adult patients with urinary tract infections (UTI) being hospitalized to the Borg Elarab district in Alexandria, Egypt, specimens of their urine were obtained, then categorized using the Centers for Disease Control and Prevention/National Healthcare Safety Network (CDC/NHSN) criteria as complicated urinary tract infection (UTI) cases (Horan et al. 2008). Urine samples were collected in sterile bottles and transported to microbiology laboratory within one hour in ice box. The samples were stored at 4 °C in the lab refrigerator for 24 h.

#### Bacterial strains

Urine samples were used for inoculation of the culture medium**.** 20 μL of urine samples was inoculated and streaked onto Luria–Bertani (LB) agar plates and incubated for 1–2 d at 37 °C. After incubation, every single colony was sub- cultured in nutrient broth for enrichment at 37˚C for 24 h. The microscopic examination of pure bacterial colonies were examined using a light microscope at (40X) magnification power. Further morphological identification (culture conditions, colony morphology, shape, etc.), biochemical and molecular identification of isolates were done according to conventional methods of Collee et al. (1996)**.** For all the experiments two UTIs isolates and two different microbial cultures of *K. pneumoniae* ATCC13883 and *E. coli* ATCC 25922 strains obtained from the Egyptian Microbial collection (EMCC) Cairo MIRCEN.

### Molecular identification of bacteria

#### DNA extraction

Genomic DNA of bacterial isolates were extracted using Phenol chloroform protocol. The quality and concentration of DNA was determined at OD_260_ using Nanodrop plate method (Thermo scientific, USA), and purity of DNA was assessed by measuring OD_260_/OD_280_ and OD_260_/OD_230_ ratios (White et al. 1990). Purified DNAs were transferred into new tubes and stored at -20 ˚C until processing.

#### Amplification of 16S rRNA gene

The polymerase chain reaction (PCR)-specific amplification of the 16S gene from obtained genomic bacterial DNA using 1492/27 primer barcodes. Forward and reverse primer sequences include: forward primer was 27F: AGAGTTTGATCCTGGCTCA, and reverse primer was 1492R: GGTTACCTTGTTACGACTT. The PCR was executed utilizing a 25 µL reaction volume, comprising of 1µL DNA (10 ng), 0.2 µL of Taq DNA polymerase (40 ng), 5 µL of 10 × Taq buffer (Ferments, Germany), 2.5 µL dNTPs, 2.5 µL of forward and reverse primers (10 pmol/l of each), and up to 25 µL with nuclease-free water. The PCR amplification process was carried out in a thermocycler ABI GeneAmp 9700 (Applied Biosystems, USA) under specific programmed conditions for one cycle at 94 ˚C for 5 min (initial denaturation), followed by 35 cycles of denaturation at 94 °C for 50 s, annealing at 56 °C for 60 s, and extension at 72 °C for 1 min. Finally, final extension step for 10 min at 56 °C. The target amplified bands were excised, eluted and purified using QIAGEN gel extraction kit (Sigma-Aldrich, Germany).

## DNA sequence and phylogenetic analysis of 16S gene

### Evaluation of the anti-biofilm activity of TAuNPs and NCS –TAuNPs composite

After mature biofilm formation of *K. pneumoniae* ESA254 and *E. coli* ESA253 strains were confirmed via biofilm detection via crystal violet, the excess crystal violet was washed away with ethanol. Different concentrations TAuNPs and NCS –TAuNPs conjugate (50, 100, and 150 µg/ml) in RPMI 1640 media were added to the resulting biofilms, the negative control untreated bacteria (0.9% NaCl) was compared, and chemical antibiotic was added as a positive control. The resulting biofilms were exposed to the TAuNPs and NCS –TauNPs conjugate for 24 h, with 3 replicates for each treatment. The ODs of the stained adherent *Klebsiella pneumonia* and UPEC strains were measured with a microplate reader at 570 (Baghini, et al. 2018; Thieme, et al. 2019; Manilal, et al. 2020).

#### Gene expression analysis of biofilm and virulence gene

The expression levels of seven genes; *entA-F* (enterobactin), *fimH* (type 1 fimbriae, *pgaA* (poly-β-1, 6-N-acetyl-D-glucosamine synthesis); *blaKPC* and *blaNDM (*carbapenemases), *blaCTX-M* (ESBLs), *luxS* (Quorum Sensing operon) resistance and virulent related genes linked with the biofilm, and virulence factors in *K. pneumoniae* ESA254 and *E. coli* ESA253 strains (Supp 1) were carried out using Real-time PCR (qRT-PCR) analysis, The β-Actin was used as a housekeeping gene for the normalization of expression of all genes. For the isolation of mRNA, the cell culture (10^7^ CFU.mL^−1^) of *K. pneumoniae* ESA254 and *E. coli* ESA253 strains (OD_650_) was incubated along with TAuNPs and NCS –TAuNPs composite at an optimal concentration (50, 100 and 150 μg/mL) AuNPs dissolved in 1.0% DMSO under shaking conditions overnight at 37 °C. The expression of the selected genes in untreated mature bacterial biofilm served as the negative control (Lee et al 2011). A total of 4 ml of bacterial cell cultures were harvested by centrifugation at 15,000 rpm for 20 min, and the obtained pellet was utilized for the isolation of the mRNA via a total RNA extraction kit (Sigma‒Aldrich). After the purification process, the RNA was analyzed on a 1% agarose gel, and its amount was determined via spectrophotometry. The complementary DNA (cDNA) was synthesized according to cDNA Synthesis Kit (Sigma‒Aldrich) protocol, the reaction mixture included 10 µg (μg) of RNA, with 5 µg (μg) being reverse transcribed. The mixture consisted of 10 ml/μL oligo dT primer, 2.5 μL 5X buffer, 2.5 μL MgCl_2_, 2.5 μL 2.5 mM dNTPs, 4 μL oligo dT, 0.2 μL 5 units/μL reverse transcriptase from Promega, Germany, and 2.5 μl RNA. The thermal cycler PCR was set to perform RT‒PCR amplification at 42 °C for a duration of 1 h, followed by incubation at 72 °C for 20 min. The Rotor-Gene 6000 system, an advanced technology developed in Germany, was employed for conducting real-time PCR analysis. The analysis required the use of 1.0 μL of diluted cDNA in triplicate to ensure precise and dependable outcomes. The qRT‒PCR primers used are listed in Supplementary Table S1, and the housekeeping gene β-actin was used as a reference gene. The total volume of the reaction was 20 µl. A small quantity of template, a precise quantity of SYBR Green Master Mix, reverse and forward primers, and sterile distilled water were meticulously added to the mixture. The reaction mixture consisted solely of water. The PCR conditions employed were as follows: the temperature was raised to 95 °C for 15 min and thereafter lowered to 60 °C for a period of 30 s. This sequence was repeated a total of 40 times. The CT values of the target genes were subtracted from the CT values of the β-actin gene to calculate the ΔCT values according to Schmittgen and Livak (2008) protocol using the 2^−ΔΔCt^ method to calculate the relative gene expression, as stated in their research.

### Anticancer activity of TAuNPs and NCS –TAuNPs conjugate

#### Cell culture

The selected cell lines include human breast adenocarcinoma (MCF-7) and normal cell lines. Human colorectal carcinoma (HCT-116) were supplied by the American Type Culture Collection and kept in liquid nitrogen (-180 °C) in the Tumor Biology Department, National Cancer Institute, Cairo, Egypt. The cell lines were plated in a 96-well tissue culture plate at a density of 1 X 10^5^ cells/ml (100 µL per well) and incubated at 37 °C for 24 h to form a complete layer of cells. Growth medium was removed from the 96-well plates when the cells formed a complete sheet. The cell layer was washed twice with wash media.

#### Evaluation of cytotoxicity by mtt assay

A standard MTT [3-[4, 5-dimethylthiazol-2-yl]- 2,5-diphenyltetrazolium bromide] cytotoxicity colorimetric assay was employed to determine the cytotoxic activity of different concentrations (12.5, 25, 50, 100 and 150 μg/mL) of TAuNPs and NCS–TAuNPs conjugate or fluorouracil (5-FU) standard anticancer drug following Mosmann (2020); Hamida et al. (2022) protocols against the breast cancer cell line (MCF-7) and colorectal carcinoma (HCT-116) cell line, Initially, 1 × 10^4^ cells were seeded in 96-well plates, then incubated in a CO_2_ incubator at 37 ◦C for 24 h. Six-fold dilutions of the sample concentrations to be tested were made in RPMI medium containing 2% serum (maintenance medium), and 0.1 mL of each dilution was tested in different wells, while 3 wells were left as controls and received only maintenance medium. Incubate the plate for 24 h, add 20 uL of prepared reagent MTT (5 mg/ml in PB) (BIO BASIC CANADA INC) to every well, and 5% CO_2_ shaking incubation for 2 h at 37 °C, in order to mix the MTT thoroughly in the media The MTT dye was removed, and all wells were dissolved in isopropanol. Lastly, the samples' absorbance was read using a spectrostar microplate ELISA reader (Spectrostar, Germany) at 570 nm. The cell toxicity at each concentration and half-maximal inhibitory concentration (IC_50_) were calculated using the following formula (Farmahini et al. 2022).

$$ {\text{Cell survival }}\left( {{\text{viability}}} \right) \, = \frac{{({\text{At}} - {\text{Ab}})}}{{\left( {{\text{Ac}} - {\text{Ab}}} \right)}} \, \times { 1}00\% $$

Cell inhibition = 100-cell survival.

At = Absorbance value of test compound, Ab = Absorbance value of blank, Ac = Absorbance value of control.

### Statistical analysis

The data collected throughout the study were analyzed via SPSS 24.0 software. The Wilcoxon signed-rank test, the Friedman test, and Spearman’s correlation were used to analyze the differences between the two groups. A heatmap was generated by transforming the RT‒PCR data into relative expression data. It was then displayed as a graphical representation of the differential expression of genes. The clustering procedure was performed using the average linkage and the Euclidean distances.

## Results

### Uropathogenic strains identification

The investigated bacterial strains were isolated from urine samples collected from adult patients with complicated urinary tract infection, the A urinary tract infection (UTI) bacterial isolates were identified at molecular levels by sequencing 16S gene and deposited in GenBank as *K. pneumoniae* strain ESA254 and *E. coli* strain ESA253 under accession numbers (PV017749 and PV017748), respectively.

### Characterization of uropathogenic strains

According to conventional biochemical characterization tests, both investigated Gram (-ve) bacterial isolates exhibited positive catalase activity and negative oxidase activity. UTIs *E. coli* ESA253 isolate and *E. coli* ATCC 25922 reference strain have negative results for urease, citrate utilization, and the Voges-Proskauer test, but positive results for methyl red (MR) and indole synthesis. The biochemical assays for UTIs *K. pneumoniae* ESA254 isolate and *K. pneumoniae* ATCC13883 reference strain provide good results for urease, citrate utilization, and the Voges-Proskauer test, but negative results for indole and methyl red as shown in Supplementary Table S2.

### Antibiotic susceptibility of uropathogenic strains

Analysis of the antimicrobial susceptibility pattern of uropathogenic strains; *K. pneumoniae*, and *E. coli* isolates were further tested for their susceptibility to 14 antibiotics, and showed that *K. pneumoniae* ESA254 isolate was susceptible (lowest resistance) to Ciprofloxacin, Ceftazidime and ampicillin-sulbactam, followed by Amikacin, Chloramphenicol, Gentamicin, Cefoxitin and Streptomycin, exhibited resistance to Cefixime, Azithromycin, ampicillin, ceftriaxone and tetracycline antibiotics. While *E. coli* ESA253 isolate was susceptible to Azithromycin, Gentamicin and tetracycline, followed with ciprofloxacin, Chloramphenicol, ceftriaxone and Cefoxitin, and exhibited a resistance to ampicillin-sulbactam, Cefixime, Amikacin and ampicillin antibiotics, while *K. pneumoniae* ATCC13883 reference strain was susceptible to Ampicillin-sulbactam, Ciprofloxacin, Chloramphenicol, followed with Gentamicin, Amikacin, ceftazidime and Cefoxitin, and exhibited resistance to Cefixime, Azithromycin, ampicillin, ceftriaxone and tetracycline antibiotics, The *E. coli* ATCC 25922 reference strain was susceptible to Azithromycin, Ampicillin-sulbactam, Ciprofloxacin, ceftazidime, Cefoxitin and Amikacin, and exhibited a resistance to tetracycline, Cefixime and Ampicillin antibiotics, as shown in Supplementary Table S3.

### Qualitative phytochemicals of turmeric extract

Phytochemicals screening analysis of hydro-alcoholic turmeric rhizome extract showed the presence a large group of naturally occurring non nutrient, high ratio (+ + +) of biologically active compounds of alkaloids and flavonoids, followed with moderate amounts (+ +) of terpenoids and phenolic, and a minor amount ( +) of tannins and saponins compounds, but did not have steroids.

Total phenolic and flavonoid content of turmeric extract.

Quantitative phytochemical analysis results of showed that hydro-alcoholic extract of turmeric rhizome contained (3452 mg QE/100 g DW) of total flavonoids content and (2624 mg GAE/100 g DW) of total phenolic contents.

### Characterization of compounds in turmeric extract

**Fig. 1:**
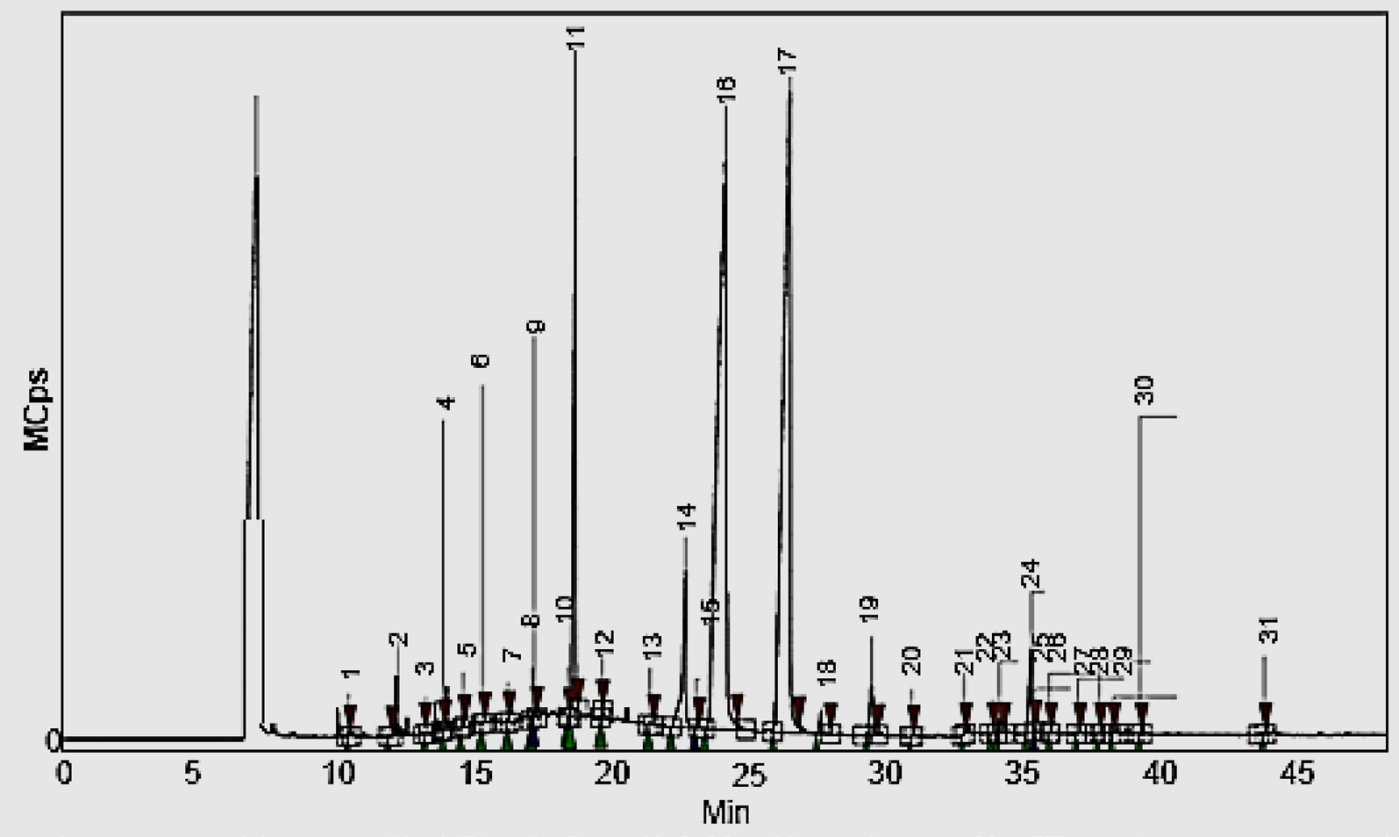
GC–MS investigation for turmeric rhizome hydroalcoholic extract

Phytochemical analysis of hydroalcholoic extract of *Curcuma longa* rhizome via GC‒MS revealed 31 bioactive components (Table 1, Fig. 1). The GC‒MS results (Fig. 1) revealed that the main remarkable constituents of turmeric extract are β-Turmerone (22.45%), Artumerone (16.68%), Zingiberene (15.1%), γ-Curcumene (10.59%), Caryophyllene (6.68%), Caryophyllene oxide (3.76%), Eucalyptol (2.28%) Coumaric acid (2.1%), Curcumin (2.096%) and Tumerone (2.09%), Sabinene (1.53%) and other bioactive components. Turmeric hydro-alcoholic extract has been used as a green approach to reduce the AuNPs ions and coated with chitosan nanoparticles producing NCS-TAuNPs for several pharmacological purposes as antibacterial and anti-cancer agents. Our result are in agreement with Moussa et al. 2023, reported that, oxygenated monoterpenes: α-turmerone (28.02%), Zingiberene (10.37%),β-turmerone (9.85%) ar-turmerone (2.61%), 1,8-cineole (6.31%), arcurcumene (8.57%), ar-curcumene (8.57%) and the α-phellandrene (4.84%), (E)-β-caryophyllene (4.61%), caryophyllene (3.54%) and sesquiterpenes: β-sesquiphelladrene (9,98%) were dominant constituents of turmeric essential oil.

**Table 1:** GC‒MS evaluation of a methanolic extract of hydro-alcoholic turmeric rhizome extract

No	RT	Name of compound	Area (%)
1	10.42	Camphene	3.274562
2	11.905	β-Pinene	0.024518
3	13.23	α-Phellandrene	0.008678
4	13.89	Benzene, 1-ethyl-3,5-dimethyl	0.019323
5	14.61	Zingiberene	15.10245
6	15.305	α-Terpinene	0.923265
7	16.24	m-Cymene	0.940158
8	17.125	Eucalyptol	2.284817
9	17.2	Curlone	1.096981
10	18.43	p-Cymen-8-ol	0.517374
11	18.69	γ-Curcumene	10.59351
12	19.67	cis-Carveol	0.904127
13	21.41	α-Curcumene	0.045546
14	22.71	Caryophyllene	6.677638
15	23.09	β-Farnesene	0.164312
16	24.165	Ar-tumerone	16.67951
17	26.51	β-Turmerone	22.44736
18	27.66	Sabinene	1.528842
19	29.485	Caryophyllene oxide	3.757821
20	30.925	Demethoxycurcumin	1.101509
21	32.845	Curcumin	2.096186
22	33.87	D-Limonene	0.50423
23	34.145	Coumaric acid	2.138912
24	35.3	Tumerone	2.090772
25	35.415	Eucalyptol	0.955093
26	35.96	Terpinolene	0.001324
27	36.995	Zingiberenol	0.041869
28	37.765	Caryophyllene	2.058751
29	38.275	Curcuphenol	0.610768
30	39.24	β -Turmerone	0.210802
31	43.77	Zingiberenol isomer	1.119171

### Green synthesis of turmeric-gold nanoparticles

This study also aimed to using turmeric extract as safe reducing agent for green synthesize of turmeric- gold nanoparticles which can substitute the other toxic traditional chemical method that generate by-products that are harmful to health and the environment alike. The biosynthesis of nanoparticles using plants is not only economically feasible but also yields NPs with enhanced biocompatibility compared to those synthesized through chemical method.

### Characterization of TAuNPs

#### UV–vis spectroscopy

The green synthesis of biogenic turmeric-gold nanoparticles was accomplished by utilizing turmeric rhizome hydro-alcoholic extract, extract induced a significant color transition from pale yellow to purple, which was established by UV–Vis spectroscopy and the surface plasmon resonance (SPR) absorption spectra ranged from 250 to 875 nm (Supplementary Fig. S1), verifying that TAuNPs were formed during incubation period with a high absorbance peak at around 575 nm, showing peaks of TAuNPs, the UV–VIS spectrophotometer of biosynthesized TAuNPs using turmeric extract exhibited strong SPR absorption at 575 nm of visible range that indicting formed spherical TAuNPs shape, and the concentration of TAuNPs was estimated according to Sanim Rahman (2016) protocol using standard TAuNP solutions (Sigma Aldrich, USA), Germany) at certain concentration to create a linear wavelength and absorption vs. concentration plot, the concentration of formed TAuNP was 35 µg/mL. The results confirm that turmeric extract acts as a reducing agent and plays a role in stabilizing environmentally friendly nanoparticle synthesis, which may be due to the occurrence of phenolic compounds, flavonoids, and other bioactive constituents.

#### The FTIR spectrum analysis of taunps

**Fig. 2:**
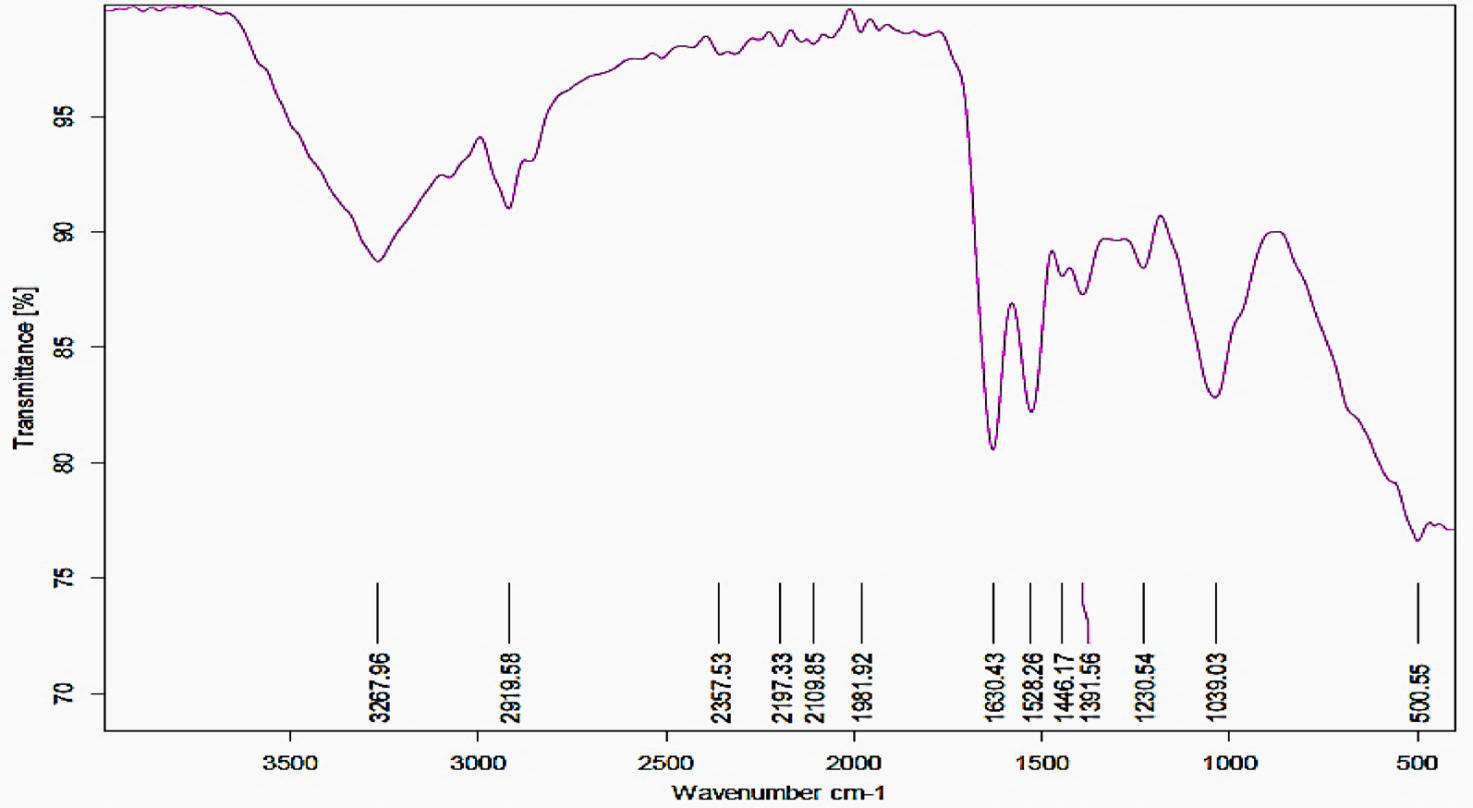
FT-IR spectroscopy of green-synthesized turmeric-gold nanoparticles from turmeric rhizome using hydro-alcoholic extract

AuNPs conjugate revealed various infrared absorption bands between 500 cm^−1^ and 3268 cm^−1^ for a series of broad peaks of functional groups at various wavenumbers (3267.8, 2919.6, 2357.5, 2197.3, 21,098.5, 1981.2, 1630.4, 1525.2, 1446.2, 1230.5, 1039, and 500.6 cm^−1^) presented at Fig. 2. The broad stretched band seen at 3267.8 cm^−1^ absorbance and the band at 2919.6 cm^−1^ can correspond to the O–H of alcohols and C-H of stretching alkynes group respectively. The broad stretched band seen at 2357.5 cm^−1^, 2197.3 cm^−1^, and 1981.9 cm^−1^ peaks correspond to the C ≡N, C = C/C≡C, and C≡C bio-functional groups, respectively. The strong broad peak at 1630.4 cm^−1^ indicates the existence of a strong carbonyl C = O group, group of amide I, and the strong broad peak at 1528.2 cm^−1^ reveals the presence of a strong carbonyl C = O group of amide II, The stretched broad band observed at 1446.2 cm^−1^, 1230.5 cm^−1^, and 1039 cm^−1^ absorbance peaks is due to the C–C, C-H, and C-N bio-functional groups, respectively.

The FTIR spectra analysis provides information on the composition and molecular structure of the TAuNPs sample analyzed, which exhibited characteristic broad O–H stretching vibrations (~ 3400 cm^−1^), C-H stretching vibrations (~ 2900 cm^−1^), and C-O stretching vibrations (~ 1100 cm^−1^) peaks are corresponding to terpenoid components of the essential oils. Stretching C-O/ C–C (~ 1644 cm^−1^), C–C (~ 1436 cm^−1^) and N–H (~ 886 cm^−1^) absorbance peaks are corresponding to monoterpenes bio functional groups. A presence of broad band O–H group at (3594.85–3262.40 cm^−1^) functional groups of turmeric rhizome extract. A Stretching O–H band occurs at (3336 cm^−1^), and stretch peaks of C-H at (2940 and 2834 cm^−1^), C = C cyclic ring at (1680–1515 cm^−1^), C-H methyl rock at 1342 cm^−1^, C-O ester at (1278 and 1126 cm^−1^) functional groups of turmeric rhizome extract. Characteristic bands at 3408, 2928, 1607 and 1314 cm^−1^ that are related to the -OH, -CH, C = C and C-O groups were confirmed, respectively. A measurement of 1021 cm^−1^ was assigned to the vibration of C-O–H distortion and 3400 to 3600 cm^−1^ to O–H stretching functional groups of turmeric rhizome extract. The analysis of FTIR spectra of gold nanoparticles indicates peaks at 3443 cm^−1^ and 1640 cm^−1^ due to the strong O–H stretch of the alcohol, and C = C variable stretch of the alkenes bioactive groups respectively, the peaks at 1544 cm^−1^ and 1384 cm^−1^ can be assigned to the N–H and N–O bending and stretching of the amide and nitro groups respectively. Other peaks at 1272 and 1206 cm^−1^ correspond to the C–O stretch of esters, and the highest intensity of the peaks confirming the crystalline nature of formed nanoparticles.

#### The transmission electron microscopy analysis of TAuNPs

**Fig. 3:**
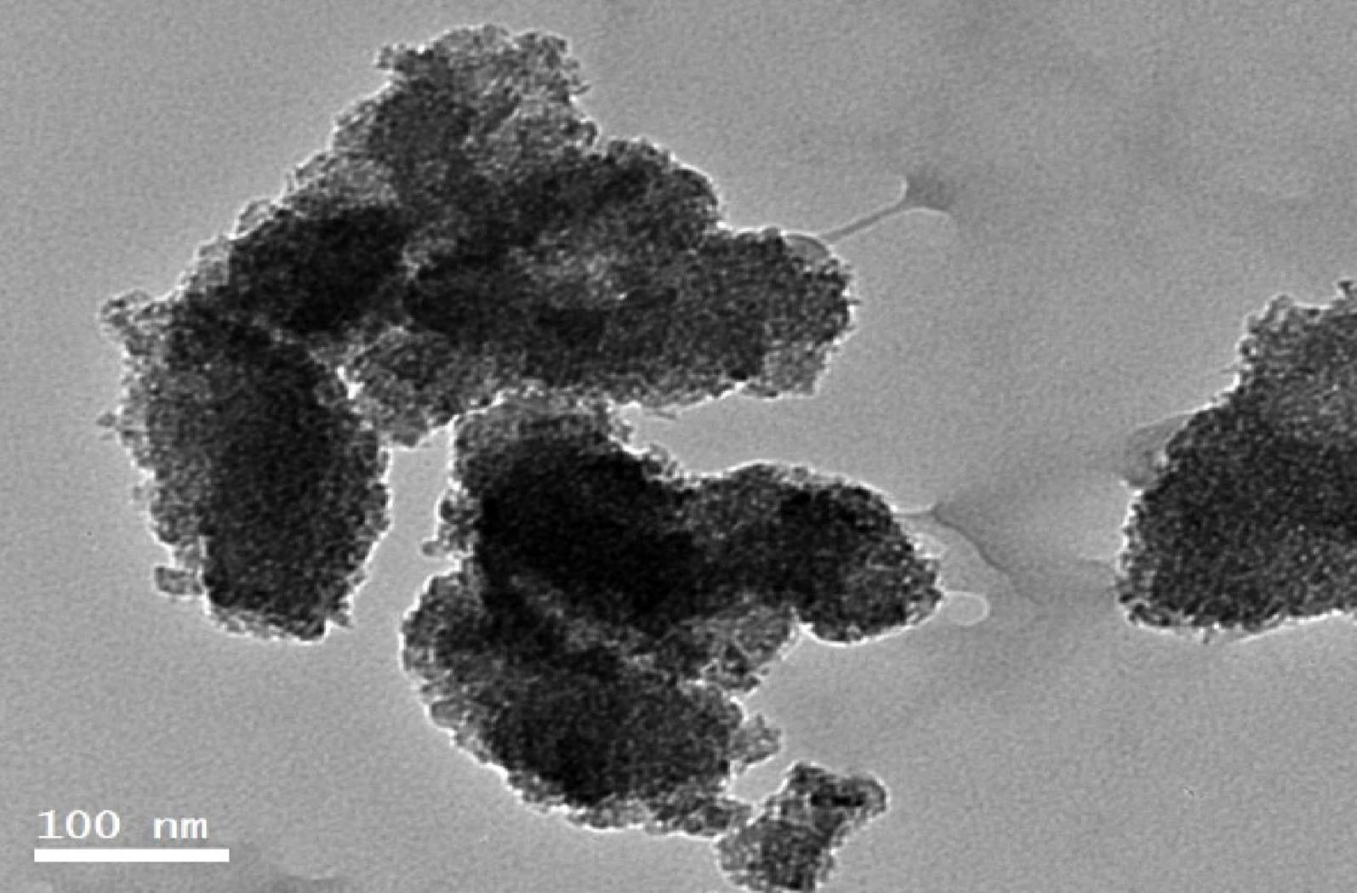
TEM graphs of green-synthesized turmeric-gold nanoparticles from turmeric rhizome using hydro-alcoholic extract

The green-synthesized turmeric- gold nanoparticles sample using turmeric rhizome extract was subjected to TEM, which is determine the real size and shape of produced nanoparticles, referred to as TAuNPs, in Fig. 3. The image revealed very small circular particles with sizes ranging from around 7–29 nm. Unfortunately, produced nanoparticles were of various shapes, such as nanospheres and nanosheets, with irregular sizes, indicating uncontrolled sizes and shape.

#### EDX spectroscopy analysis of TAuNPs

The green-synthesized turmeric-gold nanoparticles were analyzed by energy-dispersive X-ray spectroscopy (EDX). This revealed what elements are present and confirmed that gold ions are present in the TEM-formed TAuNP nanoparticles (Supplementary Fig. S2). The EDX spectra were performed at 9.7, 11.3, and 23 keV, and revealed that the obtained TAuNPs contain of several well-defined peaks associated with the gold nanostructures (Au), which are consistent with a previous study.

#### Particle size determination of TAuNPs

There are several active compounds present in the extract of turmeric root, such as polyphenols, flavonoids, and terpenoids. These compounds are helpful in reducing, stabilizing, and capping the particles in order to form TAuNPs, and also in accelerating the synthesis of small gold nanoparticles (AuNPs) synthesized in an environmentally friendly method. For stability checking, there is the method of zeta potential analysis (Supplementary Fig. S3). This revealed that the turmeric-gold nanoparticles synthesized in an environmentally friendly method are of negative potential (pH = 7). This prevents the TAuNPs from getting clumped together and prevents the nanoparticles from sticking together in water. Agglomeration of the nanoparticles takes place when they stick together, which here would decrease their surface area and their small size, which is essential for good stability of the bio-functional TAuNPs.

## Biological prosperities of TAuNPs and NCS –TAuNPs conjugate

### Antioxidant activity of TAuNPs and NCS –TAuNPs conjugate

**Fig. 4:**
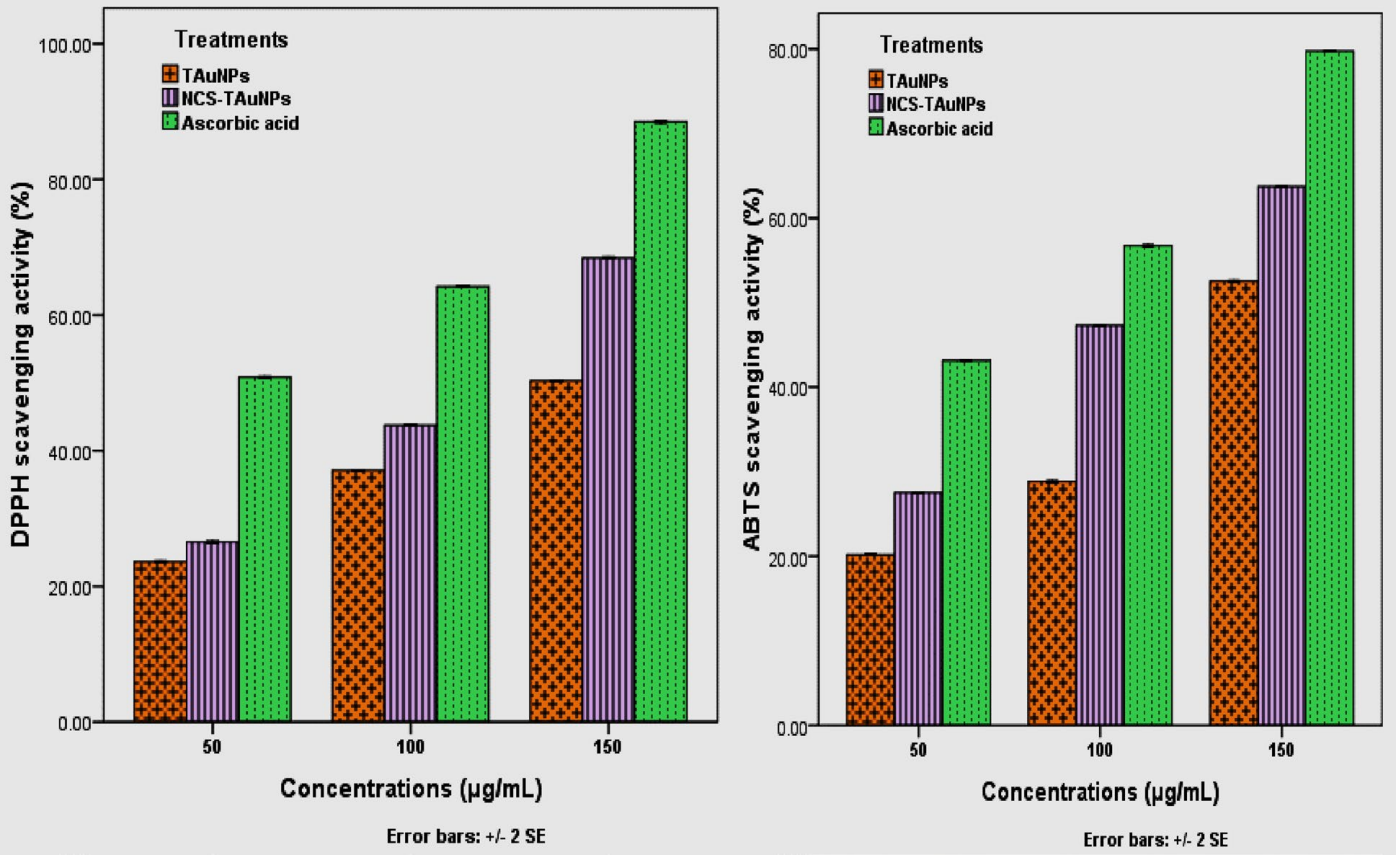
The inhibition percentage of DPPH (**a**) and ABTS (**b**) free radicals assessed with various quantities of green-made TAuNPs and NCS-TAuNPs combined, with ascorbic acid as the positive standard

The study considered the efficacy of green synthesized turmeric-gold nanoparticles (TAuNPs) and chitosan-coated turmeric-gold nanoparticles (NCS-TAuNPs) towards DPPH and ABTS free radicals. Various concentrations of TAuNPs and NCS-TAuNPs (50 µg/ml, 100 µg/ml, and 150 µg/ml), as well as 1.0% DMSO, were utilized. Following incubation, absorbance at 517 nm was measured. TAuNP and NCS-TAuNP efficacy towards DPPH and ABTS free radicals are presented in (Fig. 4A, B).

Overall, the scavenging abilities of TAuNPs and NCS-TAuNPs conjugate against ABTS free radicals were dose-dependent, a significant increase in DPPH and ABTS radical inhibition activities corresponding to an increasing the concentrations of TAuNPs and NCS-TAuNPs conjugate. The antioxidant activity results revealed that the chitosan coated turmeric-gold nanocomposite had more potential in scavenging the DPPH and ABTs free radicals than synthesized turmeric-gold nanoparticles. The maximum inhibition percentage of the DPPH radical 68.43 ± 0.095%, followed with 50.29 ± 0.038% were observed at 150 μg/mL of NCS-TAuNPs conjugate and TAuNPs respectively as compared with 88.51 ± 0.096% was recorded in 150 μg/mL ascorbic acid (positive control), while the minimum inhibition (23.63 ± 0.12%) was observed at 50 μg/mL of green-synthesized TAuNPs nanoparticles (Fig. 4A), while the maximum inhibition percentage of the ABTS radical 63.72 ± 0.09% and 52.55 ± 0.07% were recorded at 150 μg/mL of NCS-TAuNPs conjugate and TAuNPs respectively as compared with 79.74 ± 0.078%, was observed at 150 μg/mL ascorbic acid, while the minimum inhibition (20.18 ± 0.04%) was observed at 50 μg/mL of green-synthesized TAuNPs nanoparticles (Fig. 4B).

Half-maximal inhibitory concentrations (IC_50_, μg/mL) were assessed using ascorbic acid as a positive control, and the data revealed that, the IC_50_ concentrations of TAuNPs, NCS-TAuNPs conjugate and ascorbic acid for DPPH radical inhibition were 148.78 µg /mL, 105.80 µg /mL and 49.02 µg /mL respectively, while the IC_50_ values of TAuNPs, NCS-TAuNPs conjugate and ascorbic acid for inhibition ABTS free radical were 144.71 µg /mL, 115.5 µg /mL and 70.28 µg /mL, respectively.

#### Antibacterial activity of TAuNPs and NCS –TAuNPs conjugate

Antibacterial activity of TAuNPs and NCS –TAuNPs conjugate against two urinary tract infection Gram-negative bacteria isolates; *K. pneumoniae* ESA254 and *E. coli* ESA253, and *K. pneumoniae* ATCC13883 and *E. coli* ATCC 25922 reference strains were assessed in vitro using different concentrations of TAuNPs and NCS –TAuNPs composites by agar diffusion methods according to US CLSI (Clinical and Laboratory Standards Institute) protocol, The TAuNPs and NCS-TAuNPs were blended with 0.5% dimethyl sulfoxide (DMSO, Sigma-Aldrich, USA), and various concentrations (50, 100, and 150 µg/mL) of nanoparticles were prepared in 1 mL of DMSO. Bacteria were incubated in LB medium for 2 days at 37 °C. Agar plates were prepared and seeded with 20 µL of bacteria containing approximately 10^5^ CFU/mL. The plates were dried, and filter paper discs with dimensions of 5 mm were immersed in various concentrations (50, 100, and 150 µg/mL) of nanoparticles, and commercial antibiotics were incorporated at 5 µg/mL of Augmentin and gentamicin. There were three repetitions of each type of treatment performed on each plate. All plates were incubated at 37 °C for 24 h. The clear zone around the agar well upon incubation was observed to measure millimeter (mm) with the help of a scale. Microdilutions were carried out in series to determine the minimal inhibitory concentration (MIC).

Antibacterial activity of various concentrations of TAuNPs and NCS-TAuNPs in a solution of 1% DMSO, i.e., 50 μg/mL, 100 μg/mL, and 150 μg/mL, was explored against two bacteria, which are present in human urinary tracts and are resistant to the majority of drugs, i.e., ESA254 and *E. coli* ESA253, and *K. pneumoniae* ATCC13883 and *E. coli* ATCC 25922 reference strains.. This activity was compared with that of the positive control standard antibiotic solution of penicillin/streptomycin at the concentration of 10 μg/mL, and DMSO as control. The inhibition zone size of TAuNP and NCS-TAuNP composites is presented in Supplementary Fig. S4 and Table 2. The data indicate there is an antibacterial activity against both bacteria tested. The antibacterial activity of the nanoparticles varied with the dosage; greater concentrations of TAuNP and NCS-TauNP showed greater quantities of antibacterial activity compared to the positive control.

**Table 2:** Antimicrobial activity of different concentrations (50, 100 and 150 µg/mL) of TAuNPs and NCS –TAuNPs conjugate against UTIs isolates; *K. pneumoniae* ESA254 and *E. coli* ESA253, and *K. pneumoniae* ATCC13883 and *E. coli* ATCC 25922 reference strains, compared with 10 µg/ml of Gentamycin and Augmentin antibacterial standards

Concentrations (µg/mL)		Inhibition Zone (mm)			
		*K. pneumoniae *ESA254	*E. coli *ESA253	*K. pneumoniae* ATCC13883	*E. coli *ATCC 25922
TAuNPs	50	10.4 ± 0.12	6.9 ± 0.1	10.5 ± 0.1	10.3 ± 0.11
	100	12.0 ± 0.1	9.6 ± 0.12	10.8 ± 0.12	11.4 ± 0.12
	150	14.3 ± 0.3	10.8 ± 0.23	12.9 ± 0.14	12.4 ± 0.13
NCS –TAuNPs	50	12.6 ± 0.05	11.5 ± 0.24	11.5 ± 0.11	11.5 ± 0.11
	100	14.25 ± 0.12	12.5 ± 0.06	12.4 ± 0.14	14.4 ± 0.10
	150	15.6 ± 0.2	13.3 ± 0.20	14.5 ± 0.13	15.7 ± 0.13
Gentamycin	10	16.9 ± 0.31	14.3 ± 0.12	15.1 ± 0.11	15.2 ± 0.11
Augmentin	10	16.25 ± 0.24	13.7 ± 0.13	13.8 ± 0.12	13.4 ± 0.14

Values are expressed as mean ± SE (n = 3)

In addition, *E. coli* ESA253 and *E. coli* ATCC 25922 reference strain were more resistance than *K. pneumoniae* ESA254 and *E. coli* ESA253, and *K. pneumoniae* ATCC13883 (susceptible) to the green-synthesized TAuNPs and NCS-TAuNPs conjugate. The antibacterial profiles of TAuNPs and NCS-TAuNPs against *K. pneumoniae* ESA254 strain (Table 2), showed that, the highest inhibition activities of NCS-TAuNPs and TAuNPs conjugate were 15.6 ± 0.2, 14.3 ± 0.3 mm) at 150 μg/mL of NCS-TAuNPs, and TAuNPs, respectively against *K. pneumoniae* ESA254 strain, while minimum inhibition (resistance) (10.4 ± 0.12 mm, 12.0 ± 0.1 mm), were observed at 50 μg/mL and 100 μg/mL of green synthesized TAuNPs nanoparticles, as compared with the inhibition zones (16.9 ± 0.31 mm and 16.25 ± 0.24 mm) of Gentamycin and Augmentin antibacterial standards, respectively. As shown in Table 2, the antibacterial efficiency of TAuNPs and NCS-TAuNPs against *E. coli* ESA253 is presented. The highest inhibition activities of TAuNPs and NCS-TAuNPs conjugates were 13.3 ± 0.2 mm and 12.5 ± 0.06 mm at 150 μg/mL and 100 μg/mL of NCS-TAuNPs, respectively. In contrast, the minimum inhibition (resistance) was observed at 6.9 ± 0.1 mm and 9.6 ± 0.12 mm at 50 μg/mL and 100 μg/mL of green-synthesized TAuNPs, respectively. The TAuNPs and NCS-TAuNPs showed the highest inhibition activities 14.5 ± 0.13 mm, 12.9 ± 0.14 mm against *K. pneumoniae* ATCC13883 reference strain exposed to 150 (µg/mL) of NCS-TAuNPs and TAuNPs, respectively, and 15.7 ± 0.13 mm and 12.4 ± 0.13 mm against *E. coli* ATCC 25922 reference strain exposed to 150 (µg/mL) of NCS-TAuNPs and TAuNPs respectively, These values were lower compared to the inhibition zones of gentamycin (14.3 ± 0.12 mm) and Augmentin (13.7 ± 0.13 mm), respectively. The differences in resistance/susceptible ability between *K. pneumonia* and various strains may be due to the difference in bacterial cell wall. The MIC values of TAuNPs were recorded for growth inhibition of both investigated strains and was found to be 14.6 μg/mL and 18.9 μg/mL for (*K. pneumoniae* ESA254) and (*E. coli* ESA253), while MIC values of NCS-TAuNPs conjugate was found to be 6.8 μg/mL and 12.4 μg/mL for (*K. pneumoniae* ESA254) and (*E. coli* ESA253).

#### Anti-biofilm activity of TAuNPs and NCS –TAuNPs conjugate

After mature biofilm formation of in multi-drug resistant biofilm-forming of uropathogenic strains; *K. pneumoniae* ESA254 and *E. coli* ESA253, and *K. pneumoniae* ATCC13883 and *E. coli* ATCC 25922 reference strains and detected by crystal violet assay, The anti-biofilm efficiency of different concentrations (50, 100, and 150 µg/ml) of TAuNPs and NCS–TAuNPs conjugate dissolved in 1.0% DMSO against the investigated *K. pneumoniae* ESA254 and *E. coli* ESA253 strains and TAuNPs and NCS–TAuNPs are significantly reduced the amount of preformed biofilm in the total population (CFU/mL) of *K. pneumoniae* ESA254 and *E. coli* ESA253 in a dose-dependent manner. Data presented in Table 3 indicate that the highest anti-biofilm efficiency (biofilm formation reduction) in *K. pneumoniae* ESA254 (85.67 ± 2.46%, 73.46 ± 3.71%, and 68.11 ± 2.42%) was observed when *K. pneumoniae* biofilm was treated with the NCS–TAuNPs conjugate at 150 µg/mL, 100 µg/mL, and 50 µg/mL, respectively. In contrast, the lowest anti-biofilm efficiency (40.54 ± 2.15% and 47.16 ± 3.07%) was recorded at 50 µg/mL and 100 µg/mL of TAuNPs, respectively. These values were lower compared to the 94.02 ± 3.12% biofilm formation reduction observed with 10 µg/mL gentamycin treatment.

**Table 3:** Biofilm reduction percentage (%) of UTIs *K. pneumoniae* ESA254 and *E. coli* ESA253 isolates, and *K. pneumoniae* ATCC13883 and *E. coli* ATCC 25922 reference strains obtained after 24 h of exposure to the various concentrations (50 μg/mL, 100 μg/mL and 150 μg/mL) of TAuNPs and NCS –TAuNPs conjugate, as compared with 10 μg/mL Gentamycin as a positive control and negative control (0.9% NaCl) groups, (value, N = 6)

Treatments (µg/ml)		Anti-biofilm reduction (%)			
		*K.pneumoniae* ESA254	*E.coli* ESA253	*K.pneumoniae* ATCC13883	*E. coli *ATCC
TAuNPs	50	40.54 ± 2.15	36.55 ± 1.56	34.24 ± 2.1	43.7 ± 2.64
	100	47.16 ± 3.07	42.9 ± 2.38	43.78 ± 2.56	51.20 ± 3.05
	150	63.82 ± 1.89	58.07 ± 2.13	52.17 ± 4.02	63.4 ± 2.7
NCS–TAuNPs	50	68.11 ± 2.42	62.48 ± 1.78	60.73 ± 2.44	56.3 ± 2.9
	100	73.46 ± 3.71	69.27 ± 3.40	70.6 ± 2.6	71.61 ± 4.1
	150	85.67 ± 2.46	77.50 ± 2.8	81.06 ± 5.21	79.25 ± 5.23
gentamycin	10	94.02 ± 3.12	92.68 ± 3.05	93.19 ± 4.62	91.58 ± 5.7

Expression levels of Virulence Genes of *K. pneumoniae *and *E. coli *strains

Similarly, Table 3 shows that the maximum biofilm formation reduction in *E. coli* ESA253 (77.50 ± 2.8%, 69.27 ± 3.40%, and 62.48 ± 1.78%) was observed when *E. coli* biofilm was exposed to the NCS–TAuNPs conjugate at 150 µg/mL, 100 µg/mL, and 50 µg/mL, respectively. In contrast, the lowest anti-biofilm efficiency (36.55 ± 1.56% and 42.9 ± 2.38%) was recorded at 50 µg/mL and 100 µg/mL of TAuNPs, respectively. These values were lower compared to the 92.68 ± 3.05% biofilm formation reduction observed with 10 µg/mL gentamycin treatment. While the highest anti-biofilm efficiency (biofilm formation reduction) in *K. pneumoniae* ATCC13883 reference strain (81.06 ± 5.21%, 70.6 ± 2.6%, and 60.73 ± 2.44% were observed when *K. pneumoniae* biofilm was treated with the NCS–TAuNPs conjugate at 150 µg/mL, 100 µg/mL, and 50 µg/mL, respectively. In contrast, the lowest anti-biofilm efficiency (34.24 ± 2.1% and 43.78 ± 2.56%) were recorded at 50 µg/mL and 100 µg/mL of TAuNPs, respectively. And the highest anti-biofilm efficiency (biofilm formation reduction) in *E. coli* ATCC 25922 reference strain (79.25 ± 5.23%, 71.61 ± 4.1%, and 56.3 ± 2.9% were observed when *K. pneumoniae* biofilm was treated with the NCS–TAuNPs conjugate at 150 µg/mL, 100 µg/mL, and 50 µg/mL, respectively. In contrast, the lowest anti-biofilm efficiency (43.7 ± 2.64% and 51.20 ± 3.05%) were recorded at 50 µg/mL and 100 µg/mL of TAuNPs, respectively. We conclude that there are significant reductions in biofilm formation of *K. pneumoniae* ESA254 and *E. coli* ESA253 strains exposed to different concentrations of TAuNPs and NCS–TAuNPs conjugates as potential antibacterial agents.

#### Expression levels of virulence genes of K. pneumoniae and E. coli strains

**Fig. 5:**
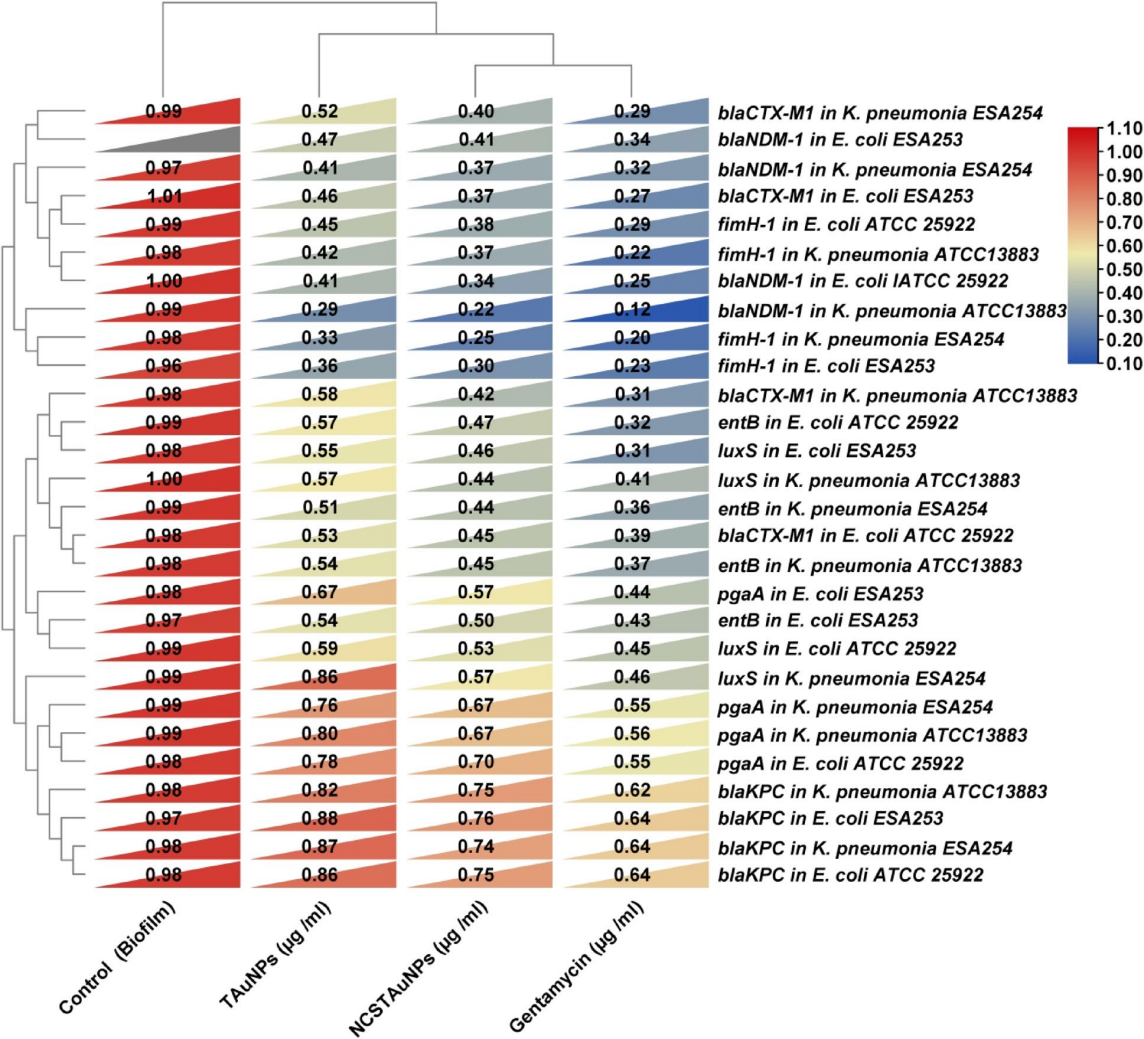
Hierarchical clustering heatmap of the transcription of relative expression of *entA-F, fimH, pgaA, blaKPC, blaNDM, blaCTX-M and luxS* resistance and virulent genes in UTIs *K. pneumoniae* ESA254 and *E. coli* ESA253 isolates, and *K. pneumoniae* ATCC13883 and *E. coli* ATCC 25922 reference strains exposed to 150 µg/ml TAuNPs and NCS–TAuNPs conjugate dissolved in 1.0% DMSO for 24 h

Overall, the reverse transcriptase analysis revealed a significant decrease in the expression profiles of virulence genes (*entB-F*, *fimH*, *pgaA*, *blaKPC, blaNDM1*, *blaCTX-M1* and *luxS*) in multi-drug resistant biofilm-forming of uropathogenic strains; *K. pneumoniae* ESA254 and *E. coli* ESA253 isolates, and *K. pneumoniae* ATCC13883 and *E. coli* ATCC 25922 reference strains biofilm treated with 150 µg/ml TAuNPs and NCS –TAuNPs conjugate in a 1.0% DMSO solution for 24 h compared with 10 μg/mL gentamycin as a positive control and negative control (0.9% NaCl) groups (Fig. 5). A hierarchical clustering heatmap of the transcription of the genes investigated is shown in Fig. 5.

The expression profile of virulent genes in *K. pneumoniae* ESA254 strain exposed to 150 µg/ml TAuNPs and NCS –TAuNPs conjugate in a 1.0% DMSO solution for 24 h (Supplementary Fig. S5A) showed that the minimum expression level (highest antibiofilm efficiency) 0.25 and 0.33 fold change of *fimH* virulence gene exposed to 150 µg/ml NCS –TAuNPs conjugate and TAuNPs nanoparticles respectively as compared with 0.2 fold change expression level in *K. pneumoniae* ESA254 isolate biofilm exposed to 10 μg/mL gentamycin positive control and 0.98 fold change in untreated *K. pneumoniae* biofilm control, followed with 0.37, 0.4, 0.44, 0.57and 0.67 and 0.74 fold changes of *blaNDM1*, *blaCTX-M, entB-F, luxS,* and *pgaA* resistance genes in *K. pneumoniae* biofilm exposed to 150 µg/ml NCS –TAuNPs conjugate respectively, while the maximum expression level and (lowest antibiofilm efficiency) 0.87 fold change of *blaKPC* virulent gene in *K. pneumoniae* biofilm exposed to 100 µg/ml TAuNPs was observed compared with positive and negative control. The minimum expression level 0.12 and 0.22 fold change of *blaNDM* virulence gene in *K. pneumoniae* ATCC13883 strain exposed to 150 µg/ml NCS –TAuNPs conjugate and gentamycin respectively, while maximum expression levels 0.82 and 0.8 folds of *blaKPC* and *pgaA* virulence genes in *K. pneumoniae* ATCC13883 strain at exposed to 150 µg/ml NCS –TAuNPs conjugate (Supplementary Fig. S5C),

The expression profile of virulence genes in *E. coli* ESA253 strain exposed to 150 µg/mL TAuNPs and NCS–TAuNPs conjugates in a 1.0% DMSO solution for 24 h (Supplementary Fig. S5B) showed that the minimum expression level (highest anti-biofilm efficiency) was observed for the *fimH* virulence gene, with 0.3- and 0.36-fold changes upon exposure to 150 µg/mL NCS–TAuNPs conjugates and TAuNPs nanoparticles, respectively. The minimum expression level 0.25 and 0.27 fold change of *blaNDM* and *blaKPC* virulence genes in *E. coli* ATCC 25922 strain exposed to 150 µg/ml NCS –TAuNPs conjugate and gentamycin respectively (Supplementary Fig. S5D), while maximum expression levels 0.86 and 0.78 folds of *blaKPC* and *pgaA* virulence genes in *E. coli* ATCC 25922 strain at exposed to 150 µg/ml NCS –TAuNPs conjugate. These values were compared to a 0.23-fold change in *K. pneumoniae* biofilm exposed to the 10 µg/mL gentamycin positive control and a 0.99-fold change in untreated *K. pneumoniae* biofilm followed with 0.37, 0.41, 0.46, 0.50 and 0.57 fold changes of *blaCTX-M, blaNDM1*, *luxS*, *entB-F*, and *pgaA* resistance genes, respectively, in *K. pneumoniae* biofilm exposed to 150 µg/mL NCS–TAuNPs conjugates. In contrast, the maximum expression level (lowest anti-biofilm efficiency) was observed for the *blaKPC* virulence gene, with a 0.67-fold change in *K. pneumoniae* biofilm exposed to 100 µg/mL TAuNPs, compared to both positive and negative controls.

#### Anticancer activity

**Fig. 6:**
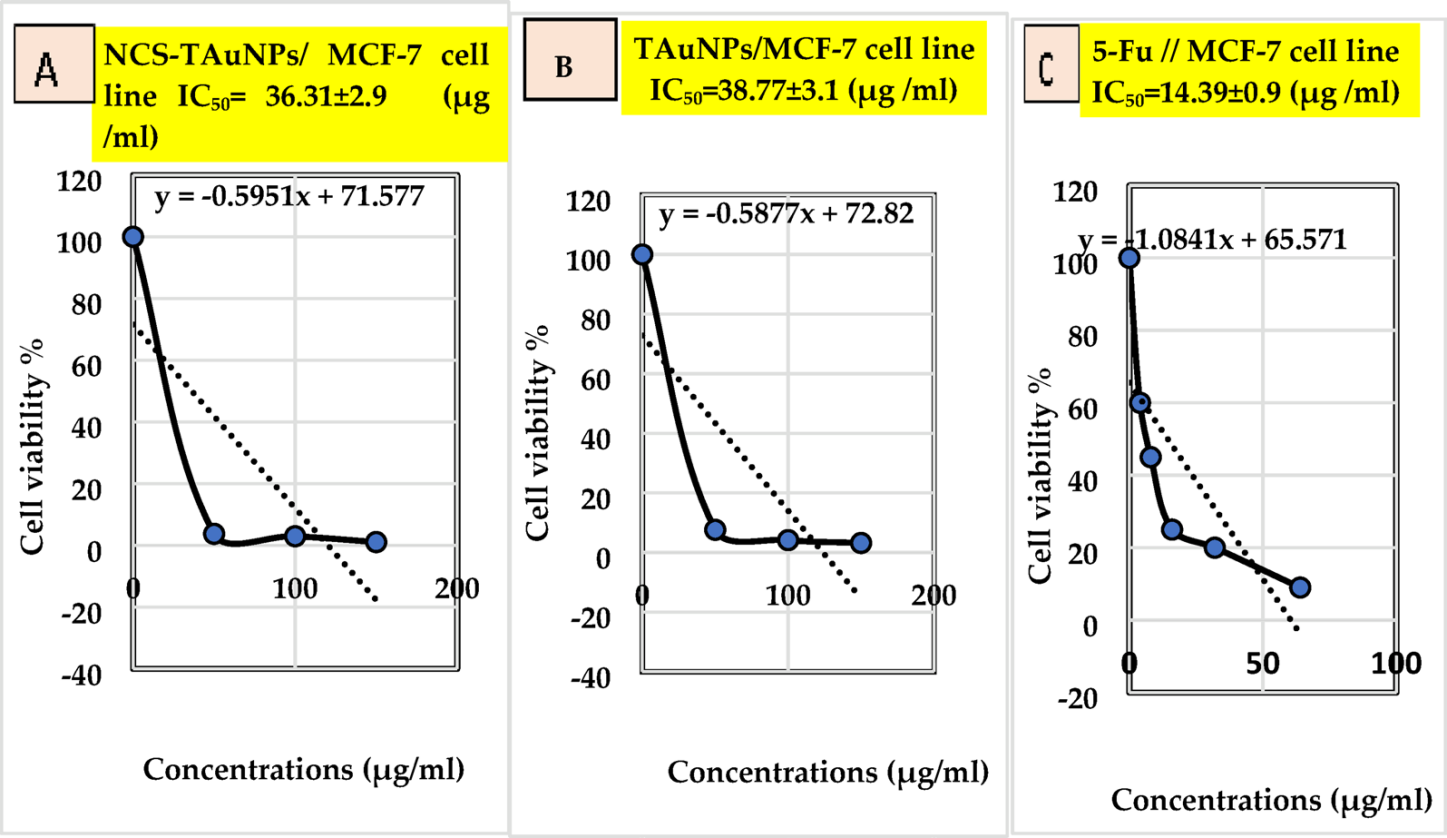
The cell viability and half-maximal inhibitory concentration (IC_50_, µg /ml) against varying amounts of NCS–TAuNPs conjugate (**a**) TAuNPs nanoparticles (**b**) the growth of MCF-7 cell lines against a negative control and 8 µg/mL of the 8 μg/mL free 5-Fu chemical anticancer drug (**C**)

**Fig. 7:**
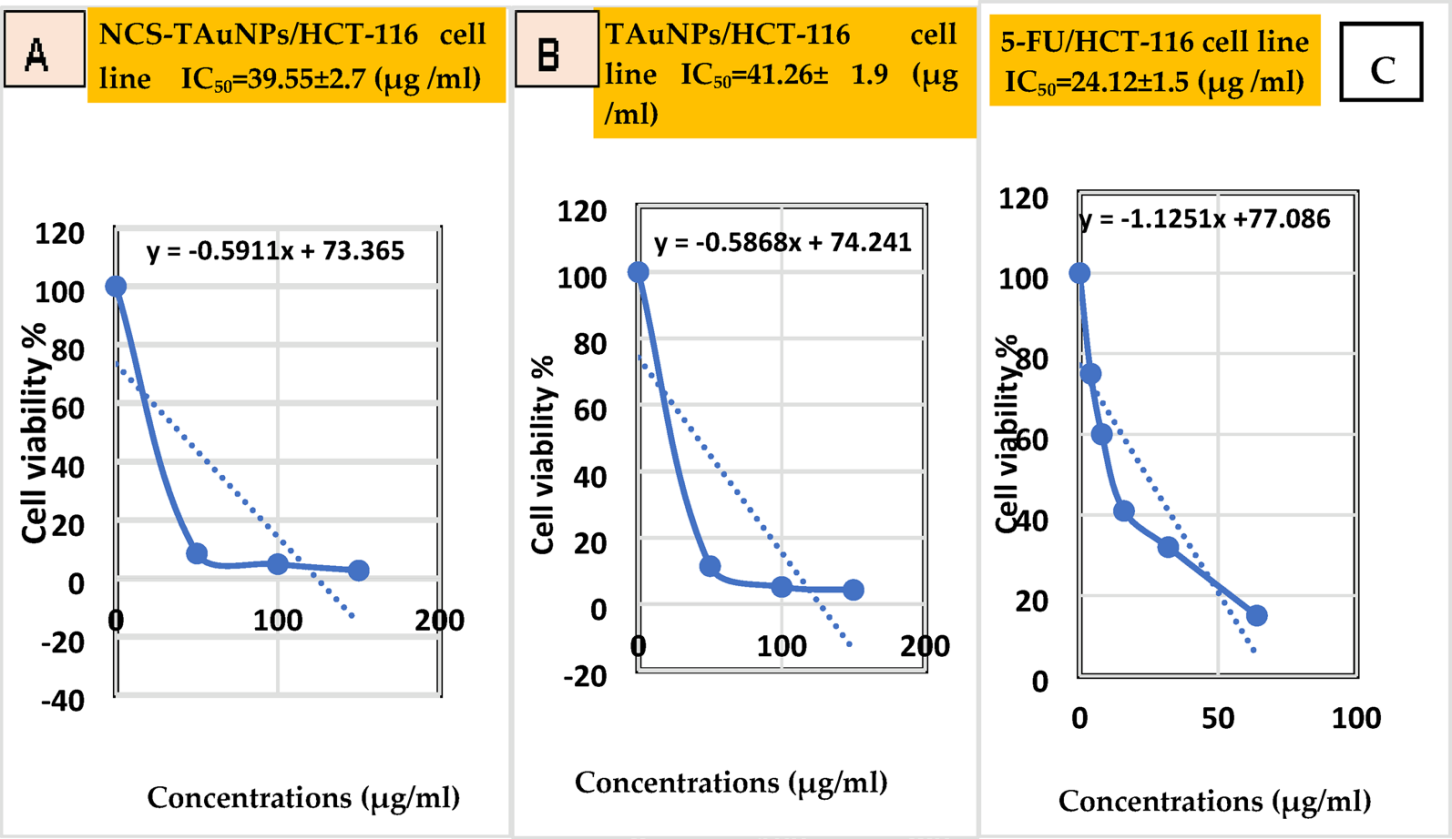
The cell viability and half-maximal inhibitory concentration (IC_50_, µg /ml) of various concentrations of NCS–TAuNPs conjugate (**a**) TAuNPs nanoparticles (**b**) and on the proliferation of HCT-116 Cell lines compared with negative control and 8 μg/mL free 5-Fu chemical anticancer drug (**C**)

The toxic effect of chitosan-coated gold nanoparticles prepared in a safe manner was investigated for its ability to inhibit growth of human breast cancer cells (MCF-7) and human colorectal cancer cells (HCT-116). Cell viability and half-maximal inhibitory concentration (IC_50_ value) were examined in cells that were treated with varying quantities (12.5 μg/mL, 25 μg/mL, 50 μg/mL, 100 μg/mL, 150 μg/mL) of these nanoparticles dissolved in 1.0% DMSO for 48 h. These were compared against a negative control and an 8 μg/mL dose of the 5-Fu drug in its pure form (positive control). The influence of these nanoparticles on breast cancer cells (MCF-7) and colorectal cancer cells (HCT-116) in a lab was investigated based on the same quantities of nanoparticles dissolved in 1.0% DMSO for 48 h. The assays employed an MTT assay in comparison with an 8 μg/mL dose of the 5-Fu cancer drug. The IC_50_ values for MCF-7 cells are illustrated in Fig. 6 and Supplementary Fig. S6. With increased quantities of NCS–TAuNPs and TAuNPs, cell viability plummeted sharply with IC_50_ values of 36.31 ± 2.9 μg/mL for NCS–TAuNPs and 38.77 ± 3.1 μg/mL for TAuNPs. These are in comparison with 14.39 ± 0.9 μg/mL for 8 μg/mL of 5-Fu drug-treated MCF-7 cells. The IC_50_ values for HCT-116 cells are illustrated in Fig. 7 and Supplementary Fig. S7. With increased quantities of NCS–TAuNPs and TAuNPs, cell viability plummeted sharply with IC_50_ values of 39.85 ± 2.7 μg/mL for NCS–TAuNPs and 41.26 ± 1.9 μg/mL for TAuNPs in comparison to 24.12 ± 1.5 μg/mL for 8 μg/mL of 5-Fu drug-treated HCT-116 cells.

## Discussion

Phytochemicals screening analysis of hydro-alcoholic extract of *curcuma longa* rhizome extract showed presence a large group of naturally occurring broad spectrum of non-nutrient and bioactive compounds included high ratio (+ + +) of biologically active compounds of alkaloids and flavonoids, followed with moderate amounts (+ +) of terpenoids and phenolic, and minor amount ( +) of tannins and saponins compounds. Quantitative phytochemical analysis results of hydro-alcoholic extract of turmeric rhizome showed that presence of (3452 mg QE/100 g DW) of total flavonoids content and (2624 mg GAE/100 g DW) of total phenolic contents, phytochemical composition profile analysis of *curcuma longa* rhizome extract via GC‒MS indicated presence many 31 bio-functional compounds, and the main remarkable constituents of turmeric extract are β-Turmerone (22.45%), Ar-tumerone (16.68%), Zingiberene (15.1%), γ-Curcumene (10.59%), Caryophyllene (6.68%), Caryophyllene oxide (3.76%), Eucalyptol (2.28%) Coumaric acid (2.1%), Curcumin (2.096%) and Tumerone (2.09%), Sabinene (1.53%) and other bioactive components. Turmeric hydro-alcoholic extract has been used as a green approach to reduce the AuNPs ions and coated with chitosan nanoparticles producing NCS-TAuNPs for several pharmacological purposes as antibacterial and anti-cancer agents. Our result are in agreement with Moussa et al. 2023, reported that, oxygenated monoterpenes: α-turmerone (28,02%), Zingiberene (10,37%),β-turmerone (9,85%) ar-turmerone (2,61%), 1,8-ceneole (6,31%), ar-curcumene (8,57%), and the α-phellandrene (4,84%), (E)-β-caryophyllene (4,61%), caryophyllene(3,54%) and sesquiterpenes: β-sesquiphelladrene (9,98%) were dominated constituents of turmeric essential oil. This study also aimed to using turmeric extract as safe reducing agent for green synthesize of turmeric-gold nanoparticles which can substitute the other toxic traditional chemical method that generate by-products that are harmful to health and the environment alike. The biosynthesis of nanoparticles using plants is not only economically feasible but also yields NPs with enhanced biocompatibility compared to those synthesized through chemical method (Chinnaperumal et al. **2023).** FTIR analysis of Turmeric gold nanoparticles has shown C (~ 1436 cm^−1^) and N–H (~ 886 cm^−1^) peaks related to monoterpenes bio functional groups, and presence of O–H group at (3594.85–3262.40 cm^−1^) functional groups of turmeric (Moussa et al. 2023). There are special groups in turmeric rhizome extract having distinct functions. There is an O–H band around 3336 cm^−1^, C-H bands around 2940 and 2834 cm^−1^, C = C bands from 1680 to 1515 cm^−1^, a C-H band around 1342 cm^−1^, and C-O bands around 1278 and 1126 cm^−1^ (Nasibi et al. 2015). There are also bands around 3408, 2928, 1607, and 1314 cm^−1^ which are conventional for -OH, -CH, C = C, and C-O groups, respectively (Taylor et al. 2002). Peaks around 1544 cm^−1^ and 1384 cm^−1^ are attributed to N–H and N–O groups, respectively (Ovais et al. 2017). Peaks around 1272 and 1206 cm − 1 are due to C–O group (Suman et al. 2014).

The nanoparticles of TAuNPs were accurately measured in size using a TEM microscope. The TEM image presents minute spherical particles measuring approximately 7–29 nm in diameter. Another study by Hamidreza and Raymond (2024), employed ginger extract in fabricating ginger-gold nanoparticles. The obtained nanoparticles were of uniform shape and size ranging from 10–20 nm in diameter that can be applied for various purposes. A study by Wu et al. (2013) synthesized *Cacumen platycladi* leaf extract-AuNPs. The resulting nanoparticles had different structures, such as nanospheres and nanoplates, and varying sizes without having much control on their shape and size. *Pogostemon benghalensis* leaf extract-AuNPs, though, have been shown by (Paul et al.^2015^) to produce nanoparticles ranging 10–50 nm in size with varying shapes (spherical, triangle, hexagonal). The EDX pattern of turmeric-gold nanoparticles synthesized in an eco-friendly manner contained clear-cut peaks corresponding to the structure of gold (Au) in TAuNPs that agrees with a previous report (Wang et al. 2012).

For assessing antioxidant capacity we evaluate the DPPH and ABTS free radicals scavenging assays, the DPPH radical inhibition IC_50_ concentrations of TAuNPs, NCS-TAuNPs conjugate and ascorbic acid were 148.78 µg /ml, 105.80 µg /ml and 49.02 µg /ml respectively, while the ABTS free radical inhibition IC_50_ values of TAuNPs, NCS-TAuNPs conjugate and ascorbic acid were 144.71 µg /ml, 115.5 µg /ml and 70.28 µg /ml respectively. The results we obtained fully agree with those of earlier research of Al-Sarraj et al. (2023)**,** reported significant antioxidant activities of green synthesized gold using *Salvia officinalis* extract and chitosan capped AuNPs at various concentrations 100, 150, 200 and 300 μg/mL. Another study reported that methanolic extracts exhibited stronger DPPH activity than ethanolic extracts of turmeric rhizome with an IC_50_ value of 17.976 ± 0.789 and 21.678 ± 1.254 µg/ml, respectively (Moussa et al. 2023**).** The study evaluated the antibacterial activity of green-synthesized turmeric extract-derived gold nanoparticles (TAuNPs) and their NCS conjugate (NCS-TAuNPs) against *Klebsiella pneumoniae* and *Escherichia coli*, common urinary tract pathogens. The antibacterial effects were concentration-dependent, with higher nanoparticle concentrations resulting in greater inhibition zones. NCS-TAuNPs exhibited stronger antibacterial activity compared to TAuNPs, though both were slightly less effective than standard antibiotics like gentamicin and Augmentin. The minimum inhibitory concentration (MIC) values for NCS-TAuNPs were lower for *K. pneumoniae* than for *E. coli*, indicating higher sensitivity of the former. The findings suggest that NCS-TAuNPs have significant potential as alternative antibacterial agents. Our antibacterial results are in agreement with those of Al-Sarraj et al. (2023) observed higher antimicrobial activity of chitosan gold nanoparticles composites from *Salvia officinalis* extract compared to biogenic synthesized gold nanoparticles (BAuNPs). Another research by Amr et al., (2022) which identified antibacterial activity of Chi/AuNPs against extended-spectrum beta-lactamase (ESBL) *Klebsiella oxytoca* ATCC 51983 and *Pseudomonas aeruginosa* MTCC1034, *Staphylococcus aureus* ATCC 25923 and *Bacillus subtilis* ATCC 6633 and The diameter of inhibition zone was 14–26 mm. previous research by Hussein et al. (2021)**,** reported that an outstanding antibacterial activity of chitosan gold nanoparticles (Cs-AuNPs), and Cs-AuNPs achieved in these investigations inhibited normal growth of two Gram-positive bacterial strains methicillin-sensitive *Staphylococcus aureus* ATCC 29213 (MSSA), methicillin-resistant *Staphylococcus aureus* ATCC 43300 (MRSA), and Gram-negative *Escherichia coli* ATCC 25922 (EC) by a range of values of MIC ranging from 16 to 32.5 µg/ml. Earlier research Yien et al. (2012); Regiel-Futyra et al. (2015); Dananjaya et al. (2020); Fuster et al. (2020) and Hussein et al. (2021) showed that biosynthesis of AuNPs in chitosan have been extensively applied for bacterial growth inhibition and bio-film. The antibacterial activity of CS-AuNPs may owe its origin due to synergic effect of Au-NPs within chitosan (Bagheri et al. 2022**)**. Also, the composition analysis of turmeric rhizome exhibited presence of Sabinene (1.52%) bioactive compounds which manifested an antibacterial activity which are in agreement with Bakkali et al. (2008) report revealed that, Sesquiterpenes in plants has a connection with a protective function of these compounds against fungi, bacteria, insects and other pests, i.e., for preservation of plants. Hassan et al. 2016 determined that the major components of a sample of *C. longa* essential oil that showed high antimicrobial and antioxidant actions were β-sesquiphellandrene, α-curcumene, and pmentha-1,4 (8)-diene. The reverse transcriptase analysis demonstrated a notable reduction in the expression levels of virulence-associated genes (*entB-F, fimH, pgaA, blaKPC, blaNDM1, blaCTX-M1, and luxS*) in multi-drug resistant, biofilm-forming uropathogenic strains: *K. pneumoniae* ESA254 and *E. coli* ESA253 following treatment with 150 µg/ml of TAuNPs and NCS-TAuNPs conjugate in a 1.0% DMSO solution for 24 h. This decrease was observed in comparison to both the positive control group treated with 10 µg/mL gentamicin and the negative control group (0.9% NaCl).

Cytotoxic effect of various concentrations of TAuNPs (12.5 μg/mL, 25 μg/mL, 50 μg/mL, 100 μg/mL and 150 μg/mL) and NCS–TAuNPs conjugate against 8 μg/mL 5-Fu chemical drug was studied in vitro against cancerous breast adenocarcinoma cells of type MCF-7 (Fig. 6) and colorectal cell lines type HCT-116 (Fig. 7) and exhibited maximum cytotoxicity in the form of apparent reduction in viability due to increasing concentrations of NCS–TAuNPs conjugate and TAuNPs and in accordance Al-Sarraj et al. (2023) study that identified cancerous type MCF-7 cancer cells as highly sensitive towards 5-Fu cytotoxic effect, 5-Fu + BAuNPs and 5-Fu + Chi/BAuNPs nanocomposite compared to non-cancerous type HFs cells which were not significantly responsive towards investigated 5-Fu + NPs cytotoxic effect. Further investigation is warranted to determine the efficacy of TAuNPs and NCS-TAuNPs against a wider panel of cancer cell lines and primary tumor cells, representing diverse cancer types and molecular profiles is needed. Also, the green synthesis approach employed for TAuNPs offers environmental benefits; however, further optimization and standardization of the biosynthesis protocol addressing also the stability of the nanocomposites over extended periods are essential for potential large-scale production and clinical translation. Addressing these limitations in future investigations will be crucial for further advancing the development of TAuNPs and NCS-TAuNPs as novel antimicrobial and anticancer agents.

## Conclusion

This study successfully developed an eco-friendly, rapid, nontoxic, and cost-effective method for synthesizing TAuNPs and NCS–TAuNPs conjugates using turmeric hydro-alcoholic extract. GC–MS analysis of the turmeric rhizome extract identified 31 bioactive phytochemical compounds.

The green-synthesized TAuNPs and NCS–TAuNPs conjugates demonstrated significant bioactive potential, particularly as potent antibacterial agents. These nanoparticles exhibited strong anti-biofilm activity against *K. pneumoniae* and *E. coli*, suggesting their potential therapeutic applications for urinary tract infections.

Additionally, cytotoxicity analysis revealed that TAuNPs and NCS–TAuNPs mixtures showed strong anticancer activities at different concentrations (12.5, 25, 50, 100, and 150 μg/mL) in comparison with the reference concentration 8 μg/mL of the anticancer drug 5-FU in vitro studies on human breast cancer (MCF-7) and colorectal cancer (HCT-116) cells.

This study suggests that green-synthesized TAuNPs and NCS–TAuNPs conjugates can be suitable candidates for designing new therapeutics against *K. pneumoniae* and *E. coli* induced urinary tract infections, as well as for breast cancer treatment in biomedical applications.

The chitosan nanoconjugate form is drug carrier and intelligent therapeutic platform possessing a unique natural properties (cationic, biocompatible) that improves the efficacy of existing anticancer and antibacterial agents (AuNPs), offering a powerful strategy in the fight against; different cancers by inhibit growth of human breast cancer cells (MCF-7) and human colorectal cancer cells (HCT-116), and infections caused by resilient pathogens like *K. pneumoniae* and *E. coli*.

## Supplementary Information

Below is the link to the electronic supplementary material.

Supplementary Material 1

## Data Availability

The derived data supporting the findings of this study are available from the corresponding author upon request. The data that support the findings of this study are available upon request from the corresponding author.

## References

[CR1] Abdelhamid HN, El-Ber HM, Metwally AA, Elshazl M, Hathout RM (2019) Synthesis of CdS-modified chitosan quantum dots for the drug delivery of Sesamol. Carbohydr Polym 214:90–99 30926012 10.1016/j.carbpol.2019.03.024

[CR2] Abdallah EM, Alhatlani BY, De Paula Menezes R, Martins CHG (2023) Back to nature: medicinal plants as promising sources for antibacterial drugs in the post-antibiotic era. Plants 12(17):3077. 10.3390/plants12173077 37687324 10.3390/plants12173077 PMC10490416

[CR3] Abdallah M, Benoliel C, Drider D, Dhulster P, Chihib NE (2014) Biofilm formation and persistence on abiotic surfaces in the context of food and medical environments. Arch Microbiol 196(7):453–472 24744186 10.1007/s00203-014-0983-1

[CR4] Al-Sarraj F, Alotibi I, Al-Zahrani M, Albiheyri R, Alghamdi MA, Nass NM, Abd-Ellatif S, Makhlof RTM, Alsaad MA, Sajer BH, Elshafie HS (2023) Green synthesis of chitosan-capped gold nanoparticles using *Salvia officinalis* extract: biochemical characterization and antimicrobial and cytotoxic activities. Molecules 28(23):7762. 10.3390/molecules28237762 38067495 10.3390/molecules28237762 PMC10707927

[CR5] Altammar KA (2023) A review on nanoparticles: characteristics, synthesis, applications, and challenges. Front Microbiol 14:1155622. 10.3389/fmicb.2023.1155622 37180257 10.3389/fmicb.2023.1155622 PMC10168541

[CR6] Amr HH, Amr MS, Omar MA, Salem SS (2022) Synthesis of chitosan-based gold nanoparticles: antimicrobial and wound-healing activities. Polymers 14(11):2293. 10.3390/polym14112293 35683965 10.3390/polym14112293 PMC9182795

[CR7] Bagheri M, Validi M, Gholipour A, Makvandi P, Sharifi E (2022) Chitosan nanofiber biocomposites for potential wound healing applications: antioxidant activity with synergic antibacterial effect. Bioeng Transl Med 7:e10254 35111951 10.1002/btm2.10254 PMC8780905

[CR8] Baghini GS, Sepahi AA, Tabatabaei RR, Tahvildari K (2018) The combined effects of ethanolic extract of *Artemisia aucheri* and *Artemisia oliveriana* on biofilm genes expression of methicillin resistant *Staphylococcus aureus*. Iran J Microbiol 10(6):417–423 30873270 PMC6414741

[CR9] Bakkali F, Averbeck S, Averbeck D, Idaomar M (2008) Biological effects of essential oils—a review. Food Chem Toxicol 46:446–475 17996351 10.1016/j.fct.2007.09.106

[CR10] Blažeković B, Vladimir-Knežević S, Brantner A, Štefan MB (2010) Evaluation of antioxidant potential of *Lavandula* x *intermedia* Emeric ex Loisel. “Budrovka”: a comparative study with *L. angustifolia* Mill. Molecules 15(9):5971–5987. 10.3390/molecules15095971 20877203 10.3390/molecules15095971 PMC6257708

[CR11] Castro J, Rosca AS, Muzny CA, Cerca N (2021) *Atopobium vaginae* and *Prevotella bivia* are able to incorporate and influence gene expression in a pre-formed *Gardnerella vaginalis* biofilm. Pathogens 10:247. 10.3390/pathogens10020247 33672647 10.3390/pathogens10020247 PMC7924186

[CR12] Chang CC, Yang MH, Wen HM, Chern JC (2002) Estimation of total flavonoid content in propels by two complementary colometric methods. JFDA 10(3):3

[CR13] Chompoosor A, Han G, Rotello VM (2008) Charge dependence of ligand release and monolayer stability of gold nanoparticles by thiols. Bioconjug Chem 19:1342–1345 18553895 10.1021/bc8000694

[CR14] Clinical and Laboratory Standards Institute (2022) Performance Standards for Antimicrobial. Disk Susceptibility Tests; Approved Standard - Twelfth Edition M02- A12. CLSI, Wayne, PA, USA

[CR15] Collee JG, Miles RS, Watt B (1996) Tests for the Identification of Bacteria. In: Collee JG, Marmion BP, Fraser AG, Simmons A (eds) Mackie & McCartney Practical Medical Microbiology, 14th edn. Churchill Livingstone, New York, pp 131–151

[CR16] Colthup N, Daly L, Wiberley S (1975) Introduction to infrared and Raman spectroscopy. Elsevier. 547'.3463, 75–1270. ISBN 0–12–182552–3

[CR17] Corbierre MK, Cameron NS, Lennox RB (2004) Polymer-stabilized gold nanoparticles with high grafting densities. Langmuir 20:2867–2873 15835165 10.1021/la0355702

[CR18] Dananjaya S, Edirisinghe S, Thao NT, Kumar RS, Wijerathna H, Mudiyanselage AY, De Zoysa M, Choi D (2020) Succinyl chitosan gold nanocomposite: Preparation, characterization, in vitro and in vivo anticandidal activity. Int J Biol Macromol 165:63–70 32971172 10.1016/j.ijbiomac.2020.09.126

[CR19] DeLong RK, Reynolds CM, Malcolm Y, Schaeffer A, Severs T, Wanekaya A (2010) Functionalized gold nanoparticles for the binding, stabilization, and delivery of therapeutic DNA, RNA, and other biological macromolecules. Nanotechnol Sci Appl 3:53–63 24198471 10.2147/NSA.S8984 PMC3781690

[CR20] Devanga Ragupathi NK, Muthuirulandi Sethuvel DP, Triplicane Dwarakanathan H, Murugan D, Umashankar Y, Monk PN, Karunakaran E, Veeraraghavan B (2020) The Influence of Biofilms on Carbapenem Susceptibility and Patient Outcome in Device Associated *K. pneumoniae* Infections: Insights Into Phenotype vs Genome-Wide Analysis and Correlation. Front in Microbiol 10.3389/fmicb.2020.591679. 10.3389/fmicb.2020.591679 PMC7767932 33381089

[CR21] Elizondo-Luevano JH, Verde-Star J, González-Horta A, Castro-Ríos R, Hernández-García ME, Chávez-Montes A (2020) In vitro effect of methanolic extract of *Argemone mexicana* against *Trichomonas vaginalis*. Korean J Parasitol 58:135–145 32418382 10.3347/kjp.2020.58.2.135 PMC7231827

[CR22] European Committee on Antimicrobial Susceptibility Testing (2022) Breakpoint tables for interpretation of MICs and zone diameters. https://www.eucast.org/fileadmin/src/media/PDFs/EUCAST_files/Breakpoint_tables/v_11.0

[CR23] Farmahini SMM, Hamdi A, Mirzaee, (2022) GC/MS analysis and phyto-synthesis of silver nanoparticles using *Amygdalus spinosissima* extract: antibacterial, antioxidant effects, anticancer and apoptotic effects. Avicenna J Med Biotechnol (AJMB) 14(3):223–232 36061132 10.18502/ajmb.v14i3.9829 PMC9376992

[CR24] Fuster E, Candela H, Estévez J, Vilanova E, Sogorb MA (2021) Titanium dioxide, but not zinc oxide, nanoparticles cause severe transcriptomic alterations in T98G human glioblastoma cells. Int J Mol Sci 22(4):2084. 10.3390/ijms22042084 33669859 10.3390/ijms22042084 PMC7923231

[CR25] Fuster M, Montalbán M, Carissimi G, Lima B, Feresin GE, Cano M, Giner-Casares J, López-Cascales J, Enriz RD, Víllora, (2020) Antibacterial effect of chitosan–gold nanoparticles and computational modeling of the interaction between chitosan and a lipid bilayer model. Nanomaterials 10:2340 33255714 10.3390/nano10122340 PMC7761461

[CR26] Goldstein JI, Newbury DE, Michael JR, Ritchie NW, Scott JHJ, Joy DC (2017) Scanning electron microscopy and X-ray microanalysis. New York, United States: Springer, 689 pages

[CR27] Hamida RS, Ali MA, Almohawes ZN, Alahdal H, Momenah MA, Bin-Meferij MM (2022) Green synthesis of hexagonal silver nanoparticles using a novel microalgae *Coelastrella aeroterrestrica* strain BA_Chlo4 and resulting anticancer, antibacterial, and antioxidant activities. Pharmaceutics. 10.3390/pharmaceutics14102002 36297438 10.3390/pharmaceutics14102002 PMC9609168

[CR28] Hamidreza K, Raymond JT (2024) Structural and antimicrobial properties of synthesized gold nanoparticles using biological and chemical approaches. Front Chem 12:1482102. 10.3389/fchem.2024.1482102 39605957 10.3389/fchem.2024.1482102 PMC11598438

[CR29] Hashem AH, Shehabeldine AM, Ali OM, Salem SS (2022) Synthesis of chitosan-based gold nanoparticles: antimicrobial and wound-healing activities. Polymers 14:2293 35683965 10.3390/polym14112293 PMC9182795

[CR30] Hassan HM, Hamed AA (2022) Chitosan silver and gold nanoparticle formation using endophytic fungi as powerful antimicrobial and anti-biofilm potentialities. Antibiotics 11:668 35625312 10.3390/antibiotics11050668 PMC9137737

[CR31] Hassan W, Gul S, Rehman S, Kanwal F, Afridi MS, Fazal H, Shah Z, Rahman A, Da Rocha JB (2016) Gas chromatography coupled with mass spectrometric characterization of *Curcuma longa*: protection against pathogenic microbes and lipid peroxidation in rat’s tissue homogenate. Pak J Pharm Sci 29(2):615–621 27087084

[CR32] Horan TC, Andrus M, Dudeck MA (2008) CDC/NHSN surveillance definition of health care-associated infection and criteria for specific types of infections in the acute care setting. Am J Infect Control 36(5):309–332. 10.1016/j.ajic.2008.03.002 18538699 10.1016/j.ajic.2008.03.002

[CR33] Hussein MAM, Grinholc M, Dena ASA, El-Sherbiny IM, Megahed M (2021) Boosting the antibacterial activity of chitosan–gold nanoparticles against antibiotic–resistant bacteria by *Punica granatum* L. extract. Carbohydr Polym 256:117498 33483025 10.1016/j.carbpol.2020.117498

[CR34] Iqbal E, Salim KA, Lim LB (2015) Phytochemical screening, total phenolics and antioxidant activities of bark and leaf extracts of *Goniothalamus velutinus* (Airy Shaw) from Brunei Darussalam. JKSUS 27:224–232. 10.1016/j.jksus.2015.02.003

[CR35] Jain S, Jain A, Jain S, Malviya N, Jain V, Kumar D (2015) Estimation of total phenolic, tannins, and flavonoid contents and antioxidant activity of *Cedrus deodara* heartwood extracts. Egypt Pharm J 14:10–14

[CR36] Kumar-Krishnan S, Prokhorov E, Hernández-Iturriaga M, Mota-Morales JD, Vázquez-Lepe M, Kovalenko Y, Sanchez IC, Luna-Bárcenas G (2015) Chitosan/silver nanocomposites: synergistic antibacterial action of silver nanoparticles and silver ions. Eur Polym J 67:242–251

[CR37] Lee JH, Cho MH, Lee J (2011) 3-indolylacetonitrile decreases *Escherichia coli* O157:H7 biofilm formation and *Pseudomonas aeruginosa* virulence. Environ Microbiol 13(1):62–73. 10.1111/j.1462-2920.2010.02308 20649646 10.1111/j.1462-2920.2010.02308.x

[CR38] Mais EA, Buthenia AH, Haider YA, Hamssa EA, Majid SJ, Suresh G, Ayman AS (2025a) Investigation the synergistic effect of chitosan and iron oxide nanoparticles against multidrug resistance Proteus mirabilis via inhibition of rsbA gene: Insilico evaluation study. Inorg Chem Commun 180(2):115130. 10.1016/j.inoche.2025.115130

[CR39] Mais EA, Kholoud KA, Nedal MF, Hayfa HA , Zainab HA and Mir WA (2025b) Colistin-Conjugated Selenium Nanoparticles: A Dual-Action Strategy Against Drug-Resistant Infections and Cancer. Pharmaceutics 17:556. 10.3390/pharmaceutics17050556 40430850 10.3390/pharmaceutics17050556 PMC12114847

[CR40] Mais EA, Noor HF, Hayfa HA and Mir WA (2025c) Biosynthesized ZnO-CuO nanocomposite forbiofilm formation of *Proteus mirabilis* upon LuxS gene expression. Inorganics 13(2):65. 10.3390/inorganics13020065

[CR41] Manilal A, Sabu KR, Shewangizaw M, Aklilu A, Seid M, Merdikios B, Tsegaye B (2020) In vitro antibacterial activity of medicinal plants against biofilm-forming methicillin-resistant *Staphylococcus aureus*: efficacy of *Moringa stenopetala* and *Rosmarinus officinalis e*xtracts. Heliyon 6(1):e03303 32051871 10.1016/j.heliyon.2020.e03303 PMC7002849

[CR42] Mohamed A. El-Tayeb, Mais E. Ahmed, Fatimah S. Alkhattaf, Eman Alhomaidi, Penislusshiyan Sakayanathan. (2025). Artemisia pallens-Mediated Manganese Nanoparticles: Apoptotic Effects on Human Epidermoid Carcinoma Cells and Their Antibiofilm Properties. *Microsc Res and Tech Early View*. 10.1002/jemt.70049 10.1002/jemt.70049 40682384

[CR43] Mosmann T (2020) Rapid colorimetric assay for cellular growth and survival: application to proliferation and cytotoxicity assays. J Immunol Methods 65:55–63 10.1016/0022-1759(83)90303-4 6606682

[CR44] Mostafa EM, Abdelgawad MA, Musa A, Alotaibi NH, Elkomy MH, Ghoneim MM, Badawy MSE, Taha MN, Hassan HM, Hamed AA (2022) Chitosan silver and gold nanoparticle formation using endophytic fungi as powerful antimicrobial and anti-biofilm potentialities. Antibiotics 11(5):668. 10.3390/antibiotics11050668 35625312 10.3390/antibiotics11050668 PMC9137737

[CR45] Moussa K, Meddah B, TirTouil A, Sonnet P (2023) Chemical Composition, Antioxidant and Antibacterial Activity of *Curcuma longa* L. Essential Oils. Egypt J Chem 66(7):283–295

[CR46] Sharifiaghdam M, Shaabani E, Asghari F, Faridi-Majidi R (2021) Chitosan coated metallic nanoparticles with stability, antioxidant, and antibacterial properties: potential for wound healing application. J Appl Polym Sci 139:51766

[CR47] Nasibi M, Mohammady M, Ashrafi A, Dehno AA, Moshrefifar M, Rafiee E, Iranian N, Refining O, Region Y (2015) Nanosized scale roughness and corrosion protection of mild steel in hydrochloric acid solution and in the presence of Turmeric (*Curcuma longa*) Extract as a green corrosion inhibitor: FTIR, polarization, EIS, SEM, EDS, AFM studies, and neural network modeling. J Adhes Sci Technol 28:2001–2015

[CR48] Ovais M, Raza A, Naz S, Islam NU, Khalil AT, Ali S, Khan MA, Shinwari ZK (2017) Current state and prospects of the phytosynthesized colloidal gold nanoparticles and their applications in cancer theranostics. Appl Microbiol Biotechnol 101(9):3551–3565. 10.1007/s00253-017-8250-4 28382454 10.1007/s00253-017-8250-4

[CR49] Panda PK, Sadeghi K, Seo J (2022) Recent advances in poly (vinyl alcohol)/natural polymer based films for food packaging applications: a review. Food Packag Shelf Life 33:100904

[CR50] Paul B, Bhuyan B, Purkayastha DD, Dey M, Dhar SS (2015) Green synthesis of gold nanoparticles using *Pogestemon benghalensis* (B) O. Ktz. leaf extract and studies of their photocatalytic activity in degradation of methylene blue. Mater Lett 148:37–40. 10.1016/j.matlet.2015.02.054

[CR51] Peeters E, Nelis HJ, Coenye T (2008) Comparison of multiple methods for quantification of microbial biofilms grown in microtiter plates. J Microbiol Methods 72:157–165. 10.1016/j.mimet.2007.11.010 18155789 10.1016/j.mimet.2007.11.010

[CR52] Punasiya R, Dindorkar G, Pillai S (2020) Antibacterial and antifungal activity of flower extract of *Murraya paniculata* L. Asian J Res Pharm Sci 10(1):17–20

[CR53] Rahman S (2016) Size and Concentration Analysis of Gold Nanoparticles with Ultraviolet-Visible Spectroscopy. Undergraduate J Math Model One + Two. 10.5038/2326-3652.7.1.4872

[CR54] Regiel-Futyra A, Kus-Liśkiewicz M, Sebastian V, Irusta S, Arruebo M, Stochel G, Kyzioł A (2015) Development of noncytotoxic chitosan–gold nanocomposites as efficient antibacterial materials. ACS Appl Mater Interfaces 7:1087–1099 25522372 10.1021/am508094e PMC4326049

[CR55] Schmittgen TD, Livak KJ (2008) Analyzing real-time PCR data by the comparative C (T) method. Nat Protoc 3(6):1101–1108. 10.1038/nprot.2008.73 18546601 10.1038/nprot.2008.73

[CR56] Shao C, Yu Z, Luo T, Zhou B, Song Q, Li Z, Yu X, Jiang S, Zhou Y, Dong W, Zhou X, Wang X, Song H (2022) Chitosan-Coated Selenium Nanoparticles Attenuate PRRSV Replication and ROS/JNK-Mediated Apoptosis in vitro. Int J Nanomedicine 7(17):3043–3054. 10.2147/IJN.S370585.PMID:35832119;PMCID:PMC9273186 10.2147/IJN.S370585 PMC9273186 35832119

[CR57] Sharma R (2005) An environmental transmission electron microscope for in situ synthesis and characterization of nanomaterials. J Mater Res 20(7):1695–1707. 10.1557/jmr.2005.0241

[CR58] Suman TY, Rajasree SR, Ramkumar R, Rajthilak C, Perumal P (2014) The green synthesis of gold nanoparticles using an aqueous root extract of *Morinda citrifolia* L. Spectrochim Acta A Mol Biomol Spectrosc 118:11–16. 10.1016/j.saa.2013.08.066 24036301 10.1016/j.saa.2013.08.066

[CR59] Tamura K, Peterson D, Peterson N, Stecher G, Nei M, Kumar S (2011) MEGA5: molecular evolutionary genetics analysis using maximum likelihood, evolutionary distance, and maximum parsimony methods. Mol Biol Evol 28(10):2731–2739 21546353 10.1093/molbev/msr121 PMC3203626

[CR60] Taylor P, Chane-ming J, Vera R, Chalchat J, Cabassu P, Chane-ming J (2002) Chemical composition of essential oils from rhizomes, leaves and flowers of *Curcuma longa* L. from Reunion Island. J Essent Oil Res 14:249–251

[CR61] Thieme L, Hartung A, Tramm K, Klinger-Strobel M, Jandt KD, Makarewicz O, Pletz MW (2019) MBEC versus MBIC: the lack of differentiation between biofilm reducing and inhibitory effects as a current problem in biofilm methodology. Biol Proced Online 21:18. 10.1186/s12575-019-0106-0 31528123 10.1186/s12575-019-0106-0 PMC6743098

[CR62] Thompson J, Gibson T, Higgins D (2018) Unit 2.3 multiple sequence alignment using Clustal W and Clustal X. Curr Prot Bioinformatics 5(1):6 10.1002/0471250953.bi0203s00 18792934

[CR63] Wang AZ, Langer R, Farokhzad OC (2012) Nanoparticle delivery of cancer drugs. Annu Rev Med 63:185–198 21888516 10.1146/annurev-med-040210-162544

[CR64] Wang H, Chen Y, Wang L, Liu Q, Yang S, Wang C (2023) Advancing herbal medicine: enhancing product quality and safety through robust quality control practices. Front Pharmacol 14:1265178. 10.3389/fphar.2023.1265178 37818188 10.3389/fphar.2023.1265178 PMC10561302

[CR65] White T, Bruns T, Lee S, Taylor J (1990) Amplification and direct sequencing of fungal ribosomal RNA genes for phylogenetics. PCR Protocols: A Guide to Methods and Applications, Academic Press, USA 18:315–322

[CR66] Wu W, Huang J, Wu L, Sun D, Lin L, Zhou Y, Wang H, Li Q (2013) Two-step size-and shape-separation of biosynthesized gold nanoparticles. Sep Purif Technol 106:117–122. 10.1016/j.seppur.2013.01.005

[CR67] Yadi M, Azizi M, Dianat-Moghadam H, Akbarzadeh A, Abyadeh M, Milani M (2022) Antibacterial activity of green gold and silver nanoparticles using ginger root extract. Bioprocess Biosyst Eng 45(12):1905–1917. 10.1007/s00449-022-02780-2 36269380 10.1007/s00449-022-02780-2

[CR68] Yien L, Zin NM, Sarwar A, Katas H (2012) Antifungal activity of chitosan nanoparticles and correlation with their physical properties. Int J Biomater 2012:632698 22829829 10.1155/2012/632698 PMC3399401

[CR69] Zengin G, Sarikurkcu C, Aktumsek A, Ceylan R (2014) *Sideritis galatica* Born: a source of multifunctional agents for the management of oxidative damage, Alzheimer’s and diabetes mellitus. J Func Foods 11:538–547

