# Supplementary material for: Antimicrobial and anti-cancer potential of turmeric synthesized AuNPs and Chitosan-AuNP nanocomposites against MDR pathogens and breast/colorectal carcinoma cells
Source: AMB Express. 2025 Nov 19;15:172. doi: 10.1186/s13568-025-01953-y (PMC12634998; doi:10.1186/s13568-025-01953-y)
Supplement: Supplementary file 1 — Supplementary Material 1 [file 13568_2025_1953_MOESM1_ESM.docx]

**Supplementary Table S1** Sequences of the oligonucleotide primers used in the qRT‒PCR analysis

| Gene name |  | Sequence |
| --- | --- | --- |
| *blaCTX-M1* | F | 5′-GGTTAAAAAATCACTGCGTC-3′ |
|  | R | 5′-TTGGTGACGATTTTAGCCGC-3′ |
| *blaKPC* | F | 5′-TGTCACTGTATCGCCGTC-3′ |
|  | R | 5′-CTCAGTGCTCTACAGAAAACC-3′ |
| *fimH-1* | F | 5´- ATGAACGCCTGGTCCTTTG - 3´ |
|  | R | 5´- GCTGAACGCCTATCCCCTGC - 3´ |
| *entB* | F | 5´- ATTTCCTCAACTTCTGGGGC - 3´ |
|  | R | 5´- AGCATCGGTGGCGGTGGTCA - 3´ |
| *pgaA* |  | 5´- GCAGACGCTCTCCTATGTC- 3´ |
|  |  | 5´- GCCGAGAGCAGGGGAATC- 3´ |
| *luxS* |  | 5´- TCATGATGAGTATATCGAAGTGCGTTCG- 3´ |
|  |  | 5´- GGATCCAGTCATGTTGATGCCAGTCTTCC- 3´ |
| *blaNDM-1* |  | 5´- GGTTTGGCGATCTGGTTTTC- 3´ |
|  |  | 5´- CGGAATGGCTCATCACGATC- 3´ |
| β-Actin | F | 5´- TGA TGC TGA CAC TGG TCA TGG TG- 3 |
|  | R | 5´- CTC TGG CAA CAT CCC AGT TGA AG- 3´ |

**entA-F* (enterobactin), *fimH* (type 1 fimbriae, *pgaA* (poly-β-1, 6-N-acetyl-D-glucosamine synthesis); *blaKPC* and *blaNDM (*carbapenemases), *blaCTX-M* (ESBLs), *luxS* (**Quorum Sensingoperon gene)**

**Supplementary Table S2** Biochemical tests used to identify UTIs isolates; *K. pneumoniae* ESA254 and *E. coli* ESA253, and *K. pneumonia* ATCC13883 and *E. coli* ATCC 25922 reference strains.

| **Characterization test** | ***K. pneumoniae* ESA254** | ***E. coli* ESA253** | ***K. pneumoniae* ATCC 13883** | ***E. coli***  **ATCC 25922** |
| --- | --- | --- | --- | --- |
| **Gram staining** | -Ve | -Ve | -Ve | -Ve |
| **Oxidase** | **-** | **-** |  |  |
| **Catalase** | **+** | **+** | **+** | **+** |
| **Indole production** | **-** | **+** | **-** | **+** |
| **Methyl red** | **-** | **+** | **-** | **+** |
| **Urease** | **+** | **-** | **+** | **-** |
| **Citrateutilization** | **+** | **-** | **+** | **-** |
| **Voges-Proskauer** | **+** | **-** | **+** | **-** |

*Results based on the types of substrate utilization: Positive (+) and negative (-).

**Supplementary Table S3**  Antibiotics susceptibility and resistance patterns of the UTIs isolates; *K. pneumoniae* ESA254 and *E. coli* ESA253, and *K. pneumonia* ATCC13883 and *E. coli* ATCC 25922 reference strains.

| **Antibiotic Disc** | **Dose (µg)** | **Inhibition zone (mm) & (Antibiotic resistance pattern )** | | | |
| --- | --- | --- | --- | --- | --- |
|  |  | ***K. pneumoniae***  **ESA254** | ***E. coli***  **ESA253** | ***K. pneumoniae* ATCC13883** | ***E. coli***  **ATCC 25922** |
| **AK** | 25 | 18 (S) | 9 (R) | 18 (R) | 10 (I) |
| **AMP** | 10 | 9 (R) | 9 (R) | 9 (R) | 8 (R) |
| **AZM** | 20 | 8 (R) | 22 (S) | 9 (R) | 21 (S) |
| **C** | 15 | 18 (S) | 18 (S) | 19 (S) | 19 (S) |
| **CAZ** | 25 | 19 (S) | 13 (I) | 18 (S) | 20 (S) |
| **CFM** | 25 | 8 (R) | 8 (R) | 9 (R) | 7 (R) |
| **CIP** | 10 | 21 (S) | 19 (S) | 19 (S) | 20 (S) |
| **CN** | 10 | 18 (S) | 20 (S) | 18 (S) | 17 (S) |
| **CRO** | 25 | 9 (R) | 18 (S) | 12 (I) | 10 (I) |
| **FOX** | 15 | 18 (S) | 18 (S) | 17 (S) | 18 (S) |
| **P** | 15 | 14 (I) | 9 (R) | 15 (I) | 15 (I) |
| **S** | 20 | 18 (S) | 13 (I) | 15 (I) | 14 (I) |
| **SAM** | 20 | 19 (S) | 7 (R) | 20 (S) | 21 (S) |
| **TE** | 30 | 9 (R) | 20 (S) | 8 (R) | 9 (R) |

**E. faecium* (EF), *E. coli* (EC), *P. aeruginosa* (PE), Susceptible (S); Intermediate (I); Resistant (R). Amikacin (AK), Ampicillin (AMP), Azithromycin (AZM), Chloramphenicol (C), ceftazidime (CAZ), Cefixime (CFM), Ciprofloxacin (CIP), Gentamicin (CN), Ceftriaxone (CRO), Cefoxitin (FOX), Pencillin G (P), Streptomycin (S), Ampicillin-sulbactam (SAM), and Tetracycline (TE) antibiotics.

.


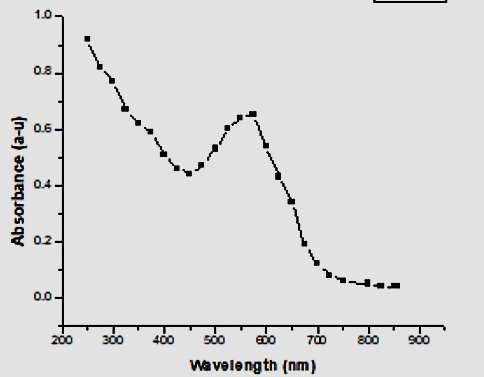


**Supplementary Fig. S1** UV-visible spectroscopy graphs of green-synthesized turmeric-gold nanoparticles from turmeric rhizome using hydro-alcoholic extracts a reducing agent, the maximum absorbance wavelength of TAuNPs preparation was at 575 nm).


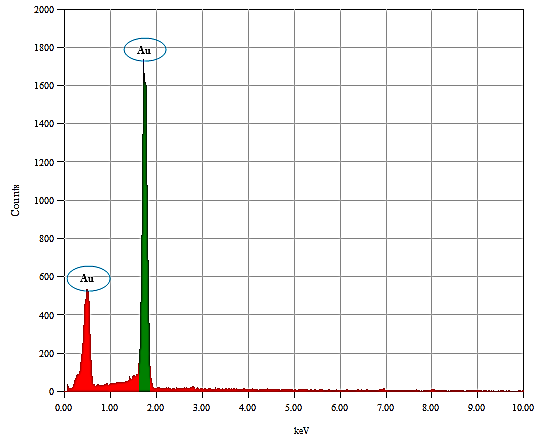


**Supplementary Fig. S2** EDX spectra reveal turmeric-gold nanoparticles derived from turmeric roots using hydro-alcoholic extract.

**
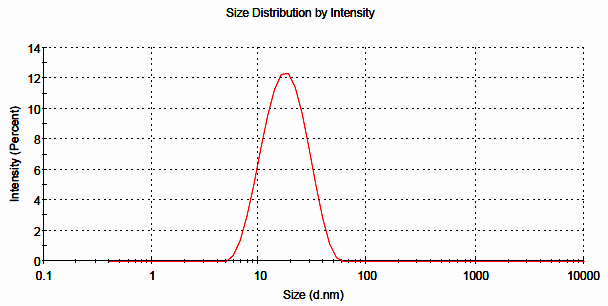
**

**Supplementary Fig. S3** Histograms of the particle sizes of the turmeric-gold nanoparticles obtained from the turmeric roots with the use of hydro-alcoholic extract.

**
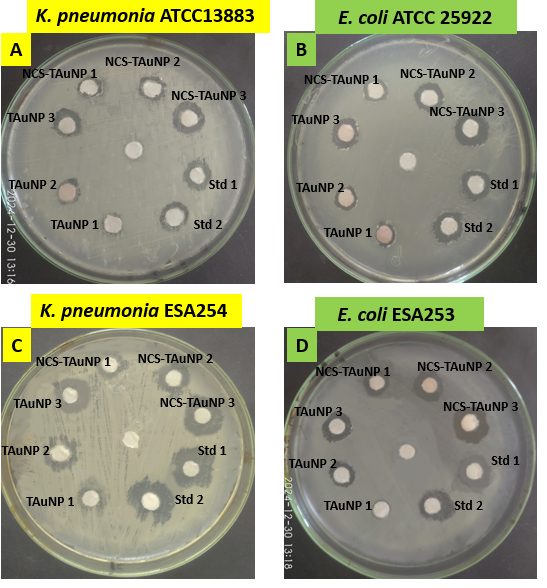
**

**Supplementary Fig. S4** showed the antibacterial activities of different concentrations ofTAuNPs and NCS –TAuNPs conjugate(50 μg/mL (TAuNPs 1 and NCS –TAuNPs 1), 100 μg/mL (TAuNPs 2 and NCS –TAuNPs 2) and 150 μg/mL (TAuNPs 3 and NCS –TAuNPs 3) against

*K. pneumonia* ATCC13883 (A) and *E. coli* ATCC 25922 (B) reference strains, and UTIs isolates; *K. pneumoniae* ESA254 (C) and *E. coli* ESA253 (D) isolates, compared with two antibacterial standards(Gentamycin 10µg/ml, Std1) and Augmentin (10µg/ml, Std2). via a filter paper diffusion assay.


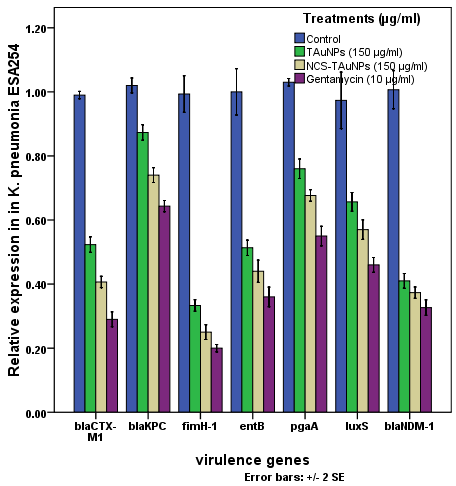

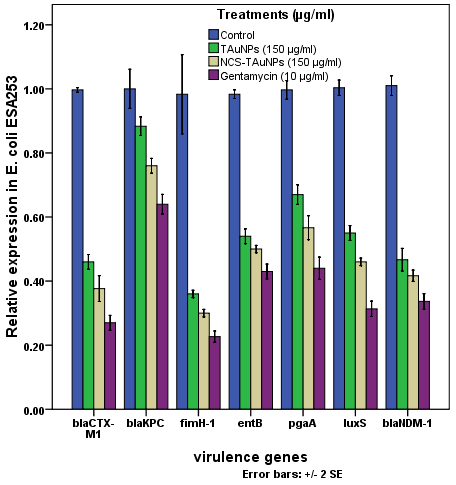


**B**

**A**


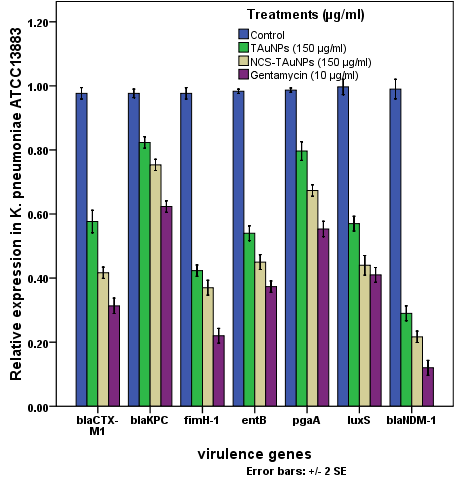

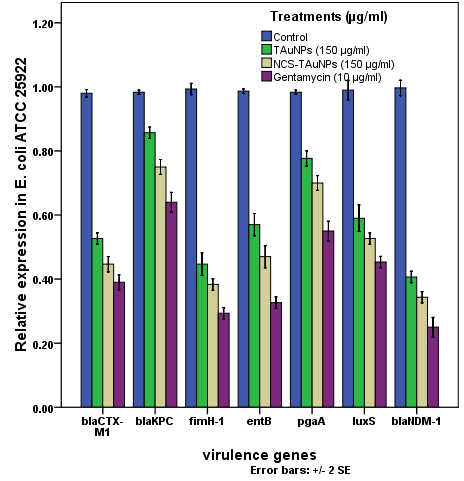


**D**

**C**

**Supplementary Fig. S5** Relative expression of entA-F, fimH, pgaA, blaKPC and blaNDM, blaCTX-M and luxS resistance and virulent genes in UTIs *K. pneumonia* ESA254 (A) and *E. coli* ESA253 (B) isolates, and *K. pneumonia* ATCC13883 (C) and *E. coli* ATCC 25922 (D) reference strains exposed to 150 µg/ml TAuNPs and NCS–TAuNPs conjugate dissolved in 1.0% DMSO for 24 h.


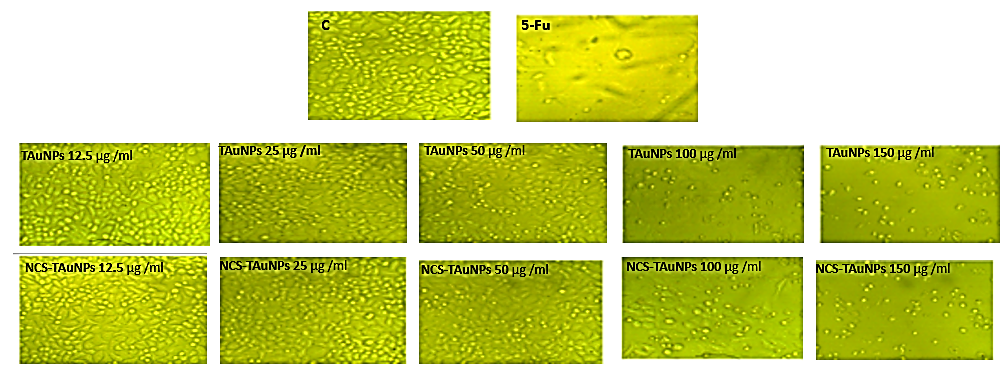


**Supplementary Fig. S6** Anticancer activity of different concentrations (12.5 μg/mL, 25 μg/mL, 50 μg/mL, 100 μg/mL and 150μg/mL) of TAuNPs and NCS–TAuNPs conjugate dissolved in 1.0% DMSO for 48h against breast cancer (MCF-7) cell line as compared with 8µg/ml Fluorouracil (5-Fu) standard.


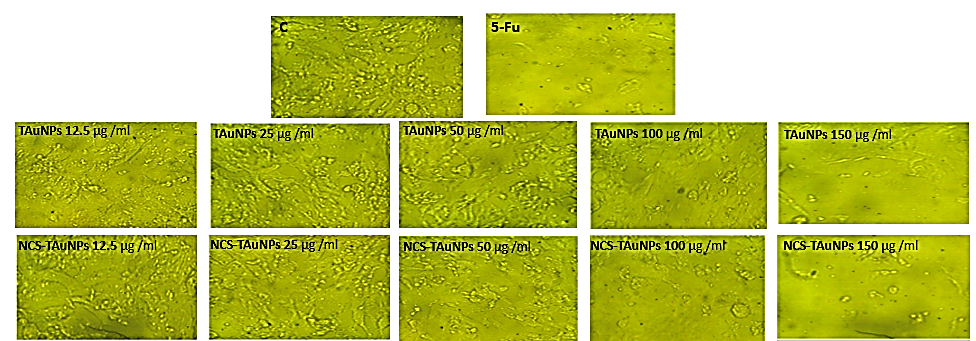


**Supplementary Fig. S7** Anticancer activity of different concentrations (12.5 μg/mL, 25 μg/mL, 50 μg/mL, 100 μg/mL and 150μg/mL) of TAuNPs and NCS–TAuNPs conjugate dissolved in 1.0% DMSO for 48h against Colorectal carcinoma (HCT-116) cell line as compared with 8µg/ml Fluorouracil (5-Fu) standard.
